# Dataset of Jaccard similarity indices from 1,597 European political manifestos across 27 countries (1945–2017)

**DOI:** 10.1016/j.dib.2019.103907

**Published:** 2019-04-09

**Authors:** William Sanger, Thierry Warin

**Affiliations:** a Polytechnique Montréal, CIRANO, Canada; b SKEMA Business School, CIRANO, Canada

**Keywords:** European elections, Data science, NLP

## Abstract

This dataset compiles the results of our computations of Jaccard similarity indices of political manifestos from 27 European countries since 1945. The raw text is accessible through the Manifesto Project's API in R. In total, 1597 political manifestos have been compared on a country level, providing measures of similarity between different political platforms. In addition to the Jaccard similarity indices gathered into 27 csv files (one per country), the R code to download, transform, and compare political manifestos is provided. Moreover, we also provide the R code to execute all the necessary computations for a whole country.

**Table:** Specifications table

Subject area	Politics
More specific subject area	Comparative politics
Type of data	27 Excel-files (.csv), 4.R code files (.R)
How data was acquired	Raw data downloaded from the Manifesto Project's API through the R library ManifestoR
Data format	Raw
Experimental factors	Original texts are transformed (uncapitalized character N-grams of 5 characters) and the methodology used is provided
Experimental features	Jaccard similarity indices are computed from uncapitalized character N-grams of 5 characters by country
Data accessibility	The data are made available in the supplementary material provided with this article

**Table:** 

**Value of the data** • The data can be used to compare how political parties present themselves through their electoral platforms. The data provide common and comparable similarity metrics across a panel of 27 European countries. This methodology is language neutral and captures the order of words in a text. • With several parties having a log time presence across numerous elections, these data can be used to track the lexical evolutions of the political parties' manifestos.

## 1 Data

The data files we provide in this article are in csv format. Each of the 27 files concerns a specific country and gathers the Jaccard similarity indices of all manifestos available from the Manifesto Project concerning a specific country. In addition to the 27 csv files, we provide four supplementary files in R format in order to reproduce all computations. Those four files help to (1) access the Manifesto Project's API (*assessManifestoText.R*), (2) transform the raw data (*transformRawData.R*), (3) compute the Jaccard similarity indice between two sets of text (*jaccardSimilarity.R*) and (4) produce all data from extracting text files from the API to producing the heatmap and csv file for one country (*completeDataForOneCountry.R*).

In recent years, the rise of populism has been noticed through some elections. More surprisingly, this dynamics has also occurred in countries with strong democratic institutions. Edgar Morin [1] even speaks of a period of historical regression. From the election of President Trump to Brexit, electoral platforms of traditional political parties (called government parties) became very close to the contesting parties, in so-called populists parties. It has become difficult to characterize far-right parties due to the diversity of movements [2], [3]. However, they could be defined by what they oppose [4] and as such, as contesting parties. They share core values, such as radicalism, populism and natalism. Radical right to far-right political parties have evolved during the past decades, with the opportunities of securing government positions in Italy (Lega) or in Austria (Freedom Party) for example.

Our motivation is the following one: how has evolved the political platform of such parties through time and across Europe? In order to answer this question, the use of raw – unedited – political platforms in their original language is particularly relevant. The Manifesto Project (https://manifesto-project.wzb.eu) collects and provides information about electoral programs aiming at comparative politics-based studies [5]. The Manifesto Project is supported by the WZB (Social Science Research Center Berlin) and DFG (German Science Foundation). Elections since 1945 from more than 50 countries are integrated into this database. Machine-readable texts are accessible through several APIs (R, Stata), as well as the manual codification of each political party's propositions. Several tools have been developed to visualise the results of these analyses as for example the evolution of political parties on the left-right axis through time. Since 2000, more than 350 refereed journal articles have been published by scholars using the Manifesto Project's database.

In our case, we use the raw text accessible through the API with the R library ManifestoR. The methodology used to compare two texts together is the Jaccard similarity indice. It is a metric ranging from [0; 1] comparing two sets of elements, such as

$$J\left(set_{1},set_{2}\right)\;=\;\frac{\left|set_{1}\cup^{}set_{2}\right|}{\left|set_{1}\cap^{}set_{2}\right|}$$

with $set_{1}$ and $set_{2}$ representing both sets of text to be compared. This metric has already been used in several fields. Historically, it originates from the botany field when, in the early 1900, Paul Jaccard quantified the number of common floral species across several sets of lands [6], [7]. It has then been used in International Business when [8], [9] used Jaccard similarity indices to measure the persistency of the TPP across free trade agreements. From a methodological perspectively, they treated text as data by using character N-grams of 5 characters before providing a coefficient of similarity. To be noticed, the use of character N-grams of 5 characters instead of bag-of-words provides the advantage of capturing the order of words in a text.

This method is interesting in the sense that it does not require the use of reference lexicons such as in sentiment analyses. This provides a greater latitude in the analysis of different languages. For instance, studies of Arabic texts [10], [11] and Thai [12] have been performed using this methodology.

In this data-based paper, we compare – at the country level – each political manifesto available from the Manifesto Project and provide the associated Jaccard indices. These metrics could serve multiple purposes, such as comparing populist manifestos to government parties’ manifestos, and evaluate this association through time across Europe.

## 2 Experimental design, materials and methods

**Fig. 1:**
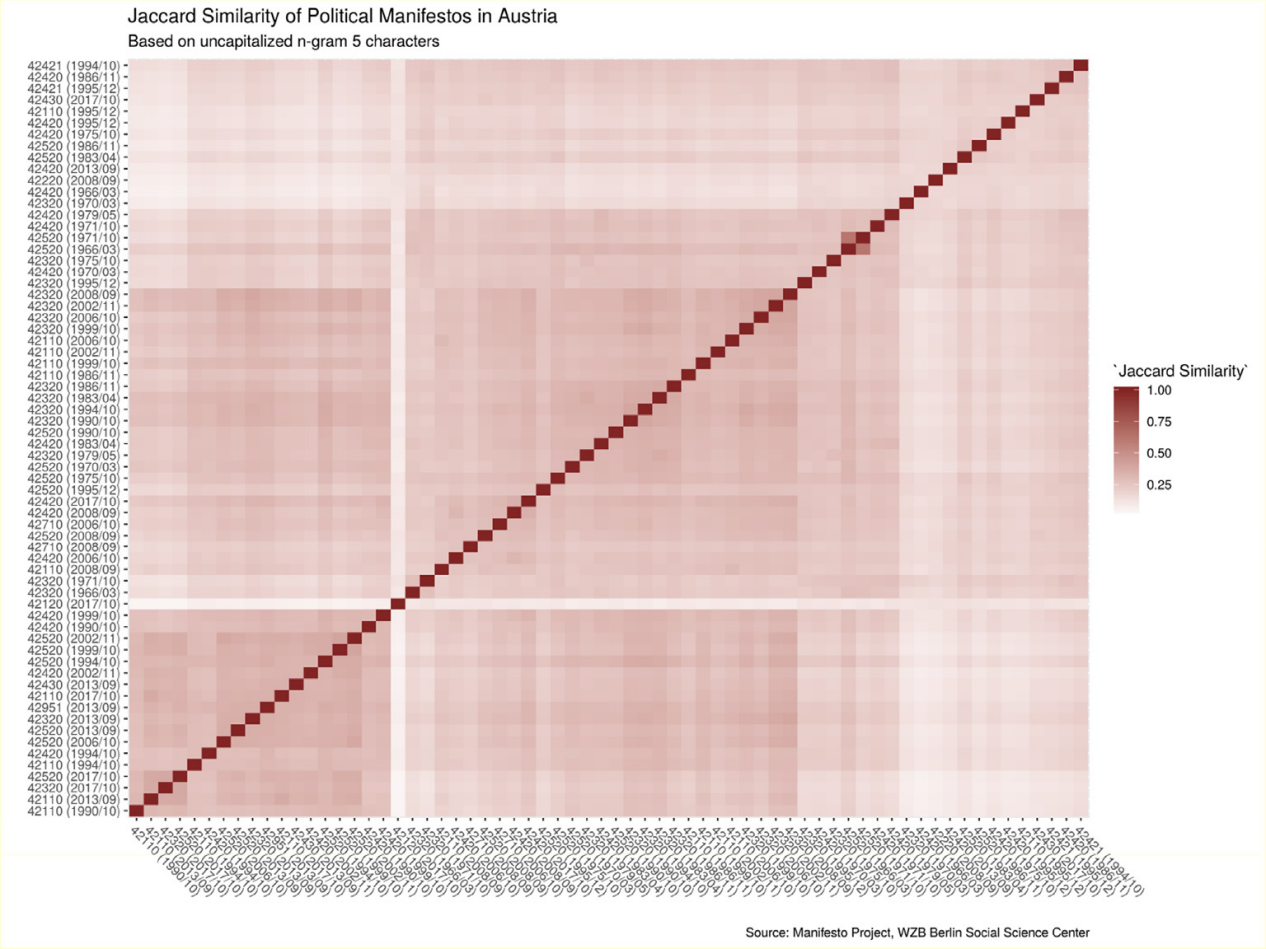
Heatmap of Jaccard indices of 66 political parties' manifestos in Austria. A possible use of the data is to track the similarity of a particular political party's manifestos through time. How was perceived one political party during its earlier elections compared to the most recent ones? illustrates this case study for the Austrian Freedom Party.

The experimental design is divided into three main parts: (1) accessing data, (2) transforming of political manifestos, and (3) comparing texts through Jaccard indices. The R code used in this article is available in the supplementary files (assessManifestoText.R, transformRawData.R, jaccardSimilarity.R, completeDataForOneCountry.R).Fig. 1

### 2.1 Accessing data

Political manifestos were obtained with the R library ManifestoR [13]. For each country, a list of available manifestos is computed. The number of political manifestos varies by country, ranging from 9 for the least (Romania) to 175 for the most (Denmark). In total, 1597 manifestos are accessible through the 2018–2 version of the Manifesto Project's database (updated on December 2018) [14].

**Fig. 2:**
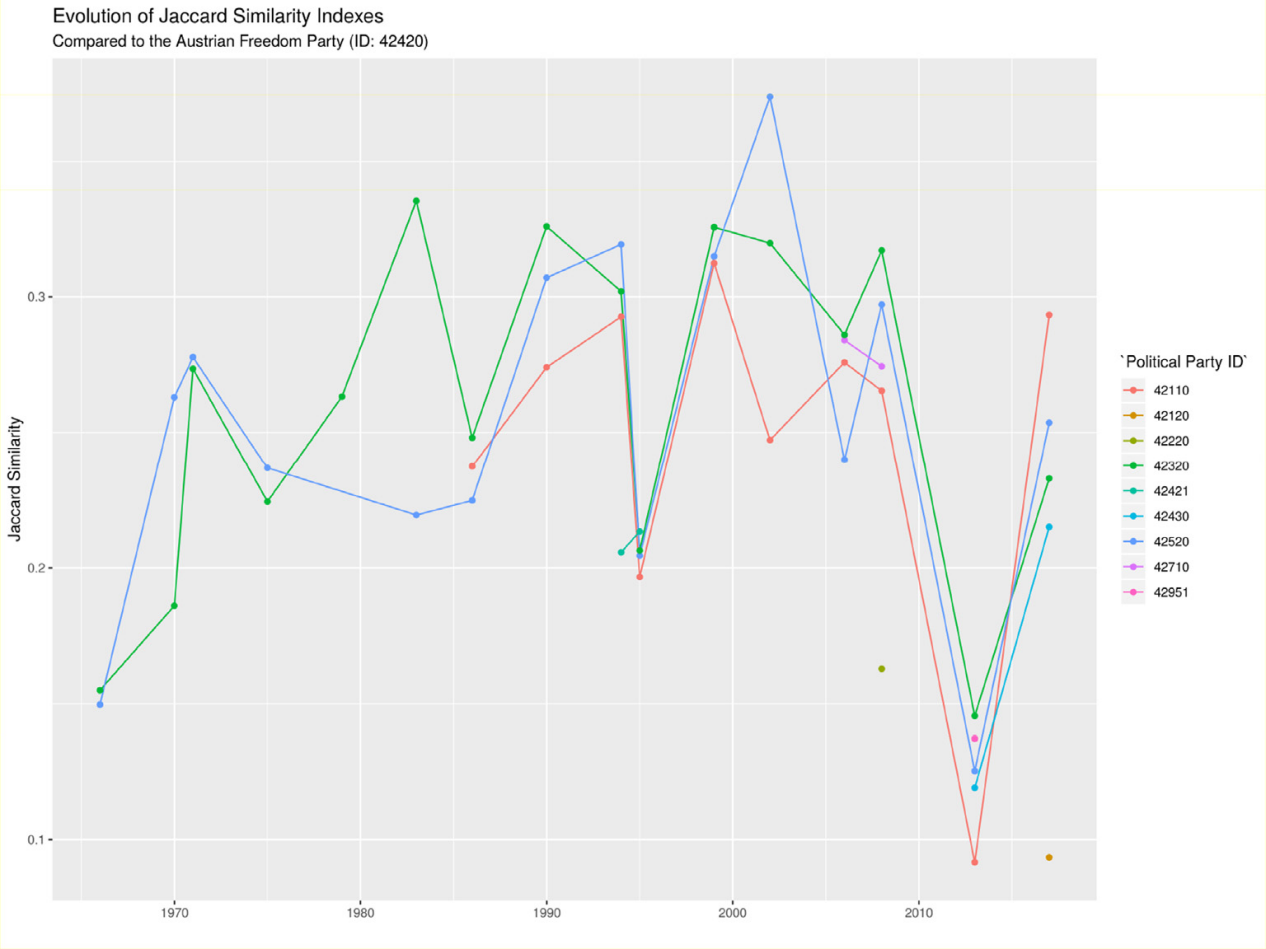
Evolution of Jaccard indices for the Austrian Freedom Party. Finally, provides an overall view of the distribution of Jaccard indices concerning the overall database (27 countries). All data is provided in the supplementary files in csv files, each file concerning a single country.

After obtaining an API key to use the Manifesto Project's API (available at: https://manifesto-project.wzb.eu/), the first step is to gather all available manifestos using the mp_corpus() function, as well as the country name and the date (after 1940) as inputs. The complete list of political parties provided by the API could be obtained using the names() function on the corpus variable previously computed. Finally, for each political manifesto, the unannotated text is considered using the content() function on the corpus of text.Fig. 2

**Fig. 3:**
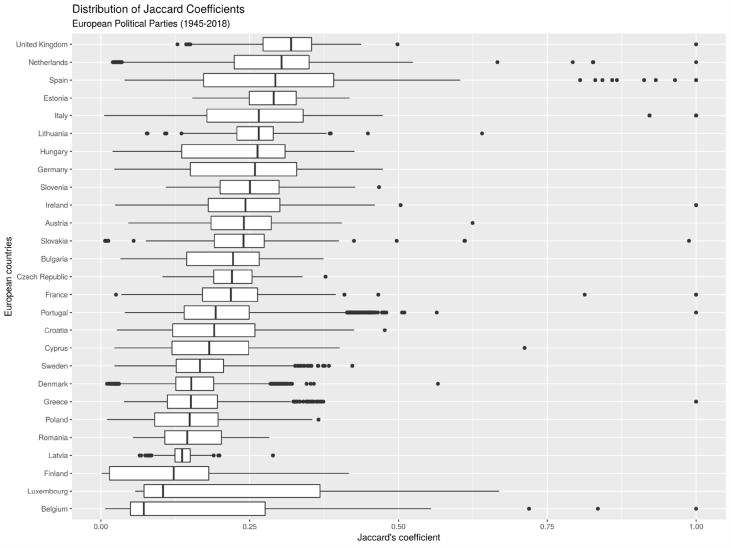
Distribution of Jaccard similarity indices across Europe.

The following table provides the number of texts associated with each country, as well as the total number of words analyzed for each country. The total number of words was assessed with the following line of R code from the stringr library in R [15]:Fig. 3^1^

**Figure:**
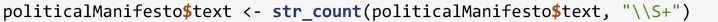

Overall, the total number of words processed to obtain the different Jaccard indices for this study is 21, 939, 796 (see Table 1).

**Table 1:** Number of political manifestos per country, as well as total number of words considered per country.

Country	Number of Political Manifestos Compared	Total Number of Words
Austria	66	689,692
Belgium	134	2,571,944
Bulgaria	18	188,564
Croatia	43	408,390
Cyprus	20	194,059
Czech Republic	31	326,416
Denmark	175	472,204
Estonia	19	140,884
Finland	97	212,468
France	65	606,740
Germany	89	1,439,767
Greece	42	570,485
Hungary	21	461,435
Ireland	75	1,131,711
Italy	100	1,171,130
Latvia	23	11,705
Lithuania	21	254,861
Luxembourg	17	548,644
Netherlands	130	2,616,752
Poland	16	241,815
Portugal	66	1,431,449
Romania	9	39,544
Slovakia	35	505,409
Slovenia	23	450,650
Spain	98	4,029,706
Sweden	103	309,794
United Kingdom	61	913,578
Complete database	1597	21,939,796

### 2.2 Data transformation

Each manifesto is then transformed before being compared to each other. First, the data type is changed to be considered as a string variable. Then, by using the tolower() function, each word is uncapitalized in order to have a uniform text. Finally, the text is divided into character N-gram of 5 characters using the substring() function. In order to account for each possible combination, the substring() function is replicated five times with a sliding selection window of one character per iteration.

**Figure:**
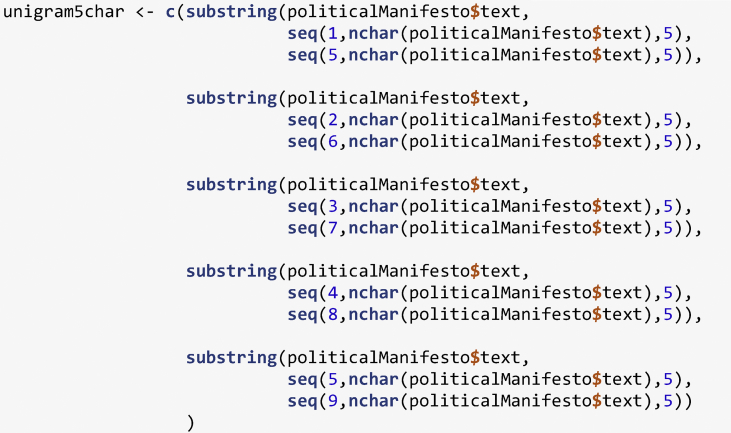

For example, the sentence “the dataset provides insight of political behaviors” would be split into: “the d”, “atas”, “et pr”, “ovide”, “s ins”, “ight”, “of po”, “litic”, “al be”, “havio”, “rs”; as well as “he da”, “taset”, " prov“,”ides “,”insig“,”ht of“,” poli“,”tical“,” beha“,”viors“,”e dat“,”aset “,”provi“,”des i“,”nsigh“,”t of “,”polit",“ical”, “behav”, “iors”, " data“,”set p“,”rovid“,”es in“,”sight“,” of p“,”oliti“,”cal b“,”ehavi“,”ors“,”datas“,”et pr“,”ovide“,”s ins“,”ight “,”of po“,”litic“,”al be“,”havio“,”rs“.

### 2.3 Jaccard indices

After transforming each political manifesto into several sets of character N-grams of 5 characters, the comparison of all political manifestos for a specific country could then be assessed. In order to do so, the function jaccard_similarity() from the textreuse library in R [16] is used. The result is an indice ranging from [0; 1], with 0 meaning that two sets of text do not share any common elements and 1 meaning that both sets share exactly the same elements. The R code line used is the following one:

**Figure:**
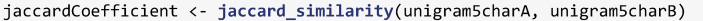

With unigram5charA and unigram5charB two sets of processed texts to be compared. These measures could illustrate how political parties promotes platform that are similar to each other. For example, concerns Austria, with a total of 60 political manifestos compared to each other. The symmetric heatmap highlights areas with stronger similarities while the lighter areas presents lower value of Jaccard indices.

## Footnotes

1 Fig. 3 provides a comparison on a country level of the distribution of Jaccard similarity indices. As such, countries can be compared one to another. However, due to different languages involved within political manifestos from different countries, two parties from two different countries are not compared.
