# Supplementary figures and images for: Dataset of Jaccard similarity indices from 1,597 European political manifestos across 27 countries (1945–2017)
Source: Data Brief. 2019 Apr 9;24:103907. doi: 10.1016/j.dib.2019.103907 (PMC6479077; doi:10.1016/j.dib.2019.103907)

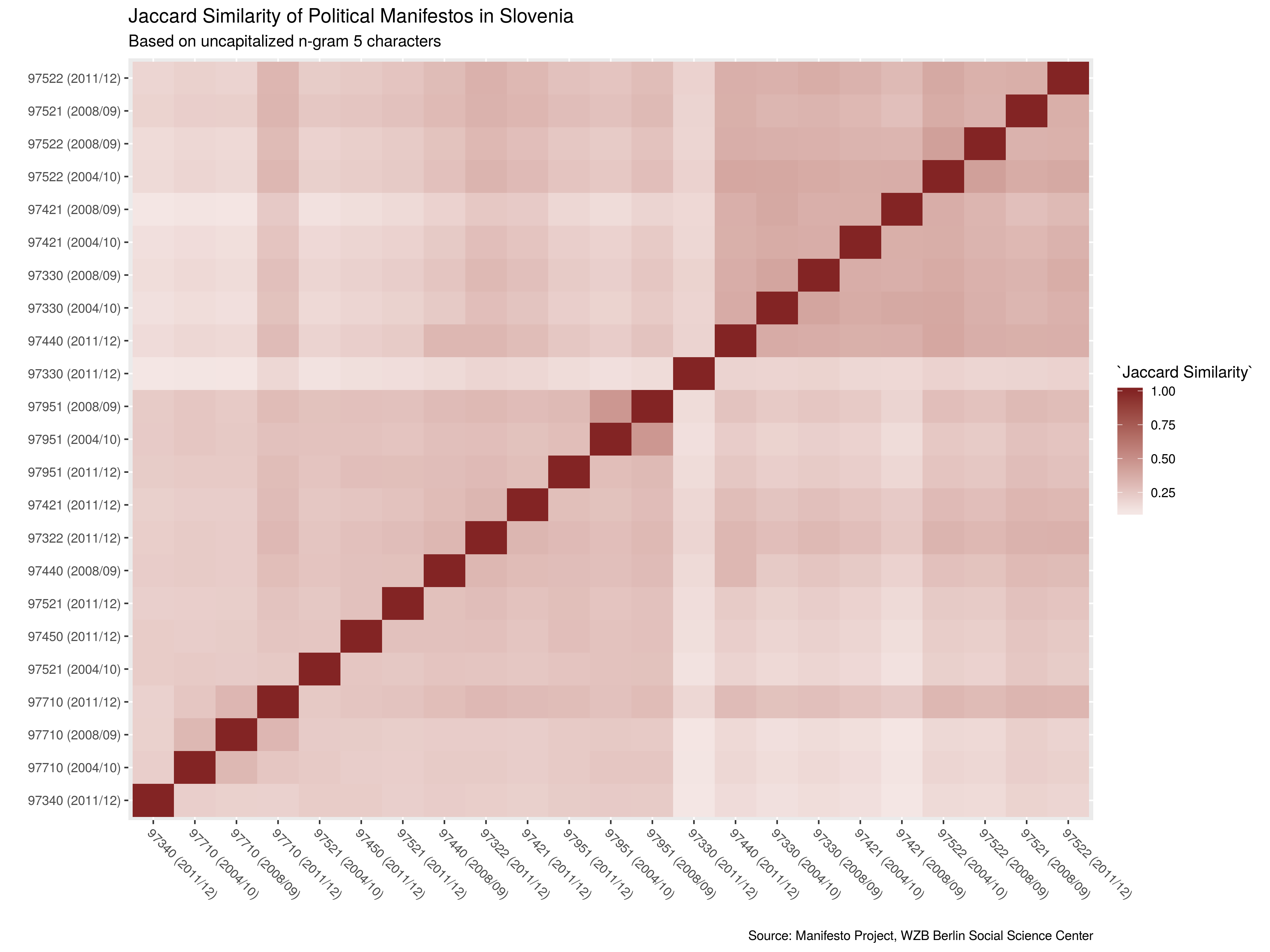

Supplement: Multimedia component 4 [file mmc4.zip › slovenia.png]

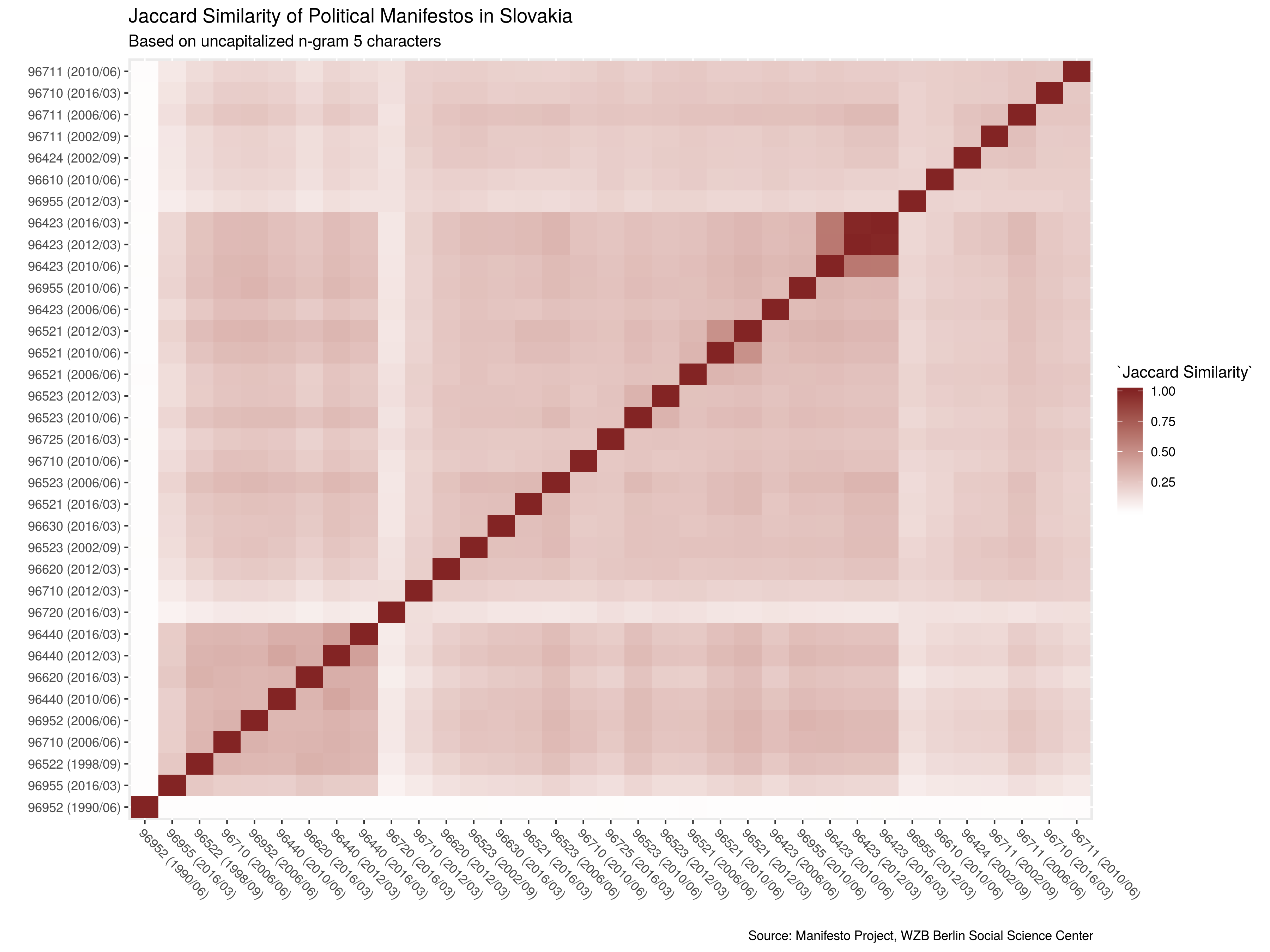

Supplement: Multimedia component 4 [file mmc4.zip › slovakia.png]

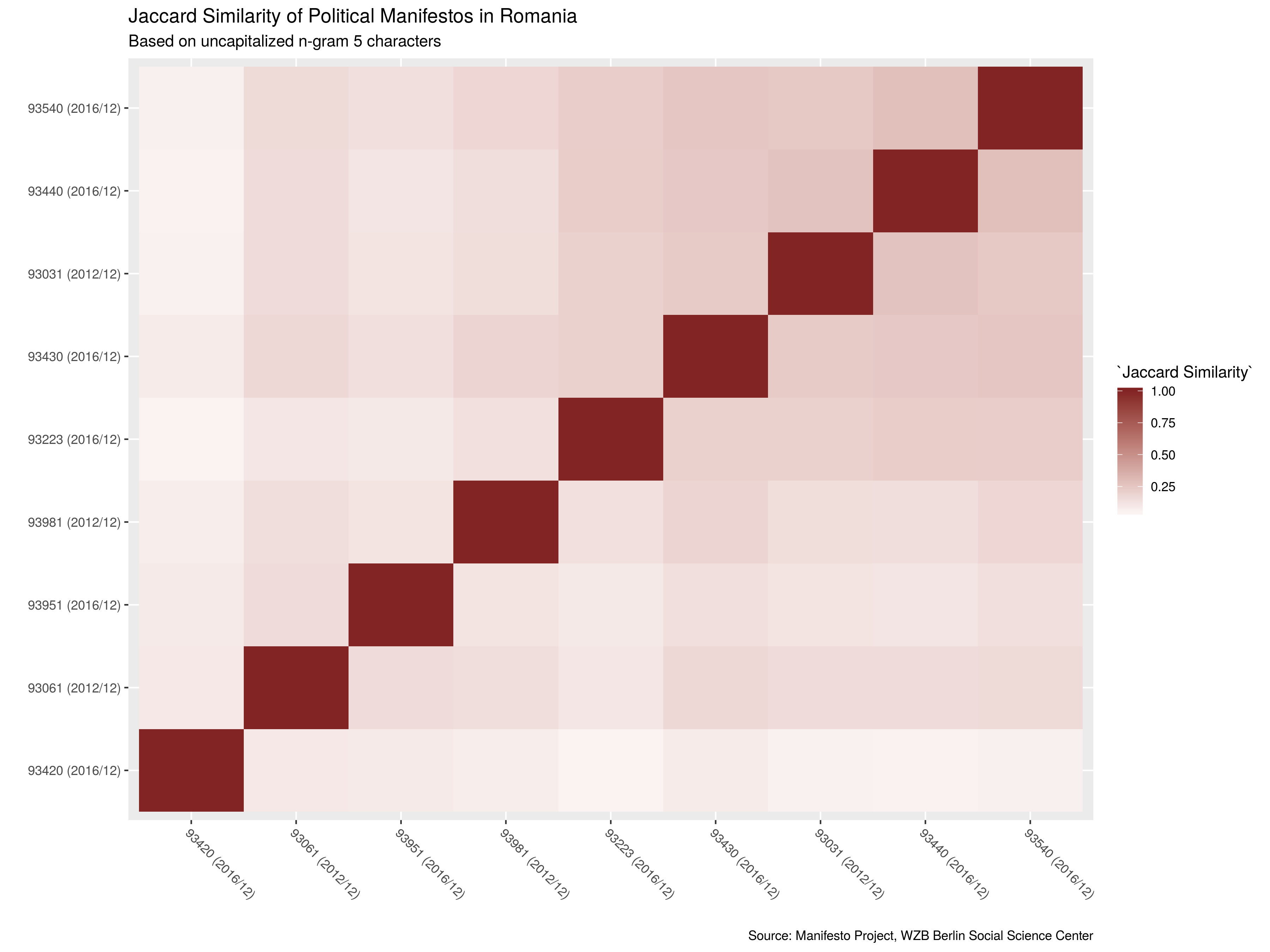

Supplement: Multimedia component 4 [file mmc4.zip › romania.png]

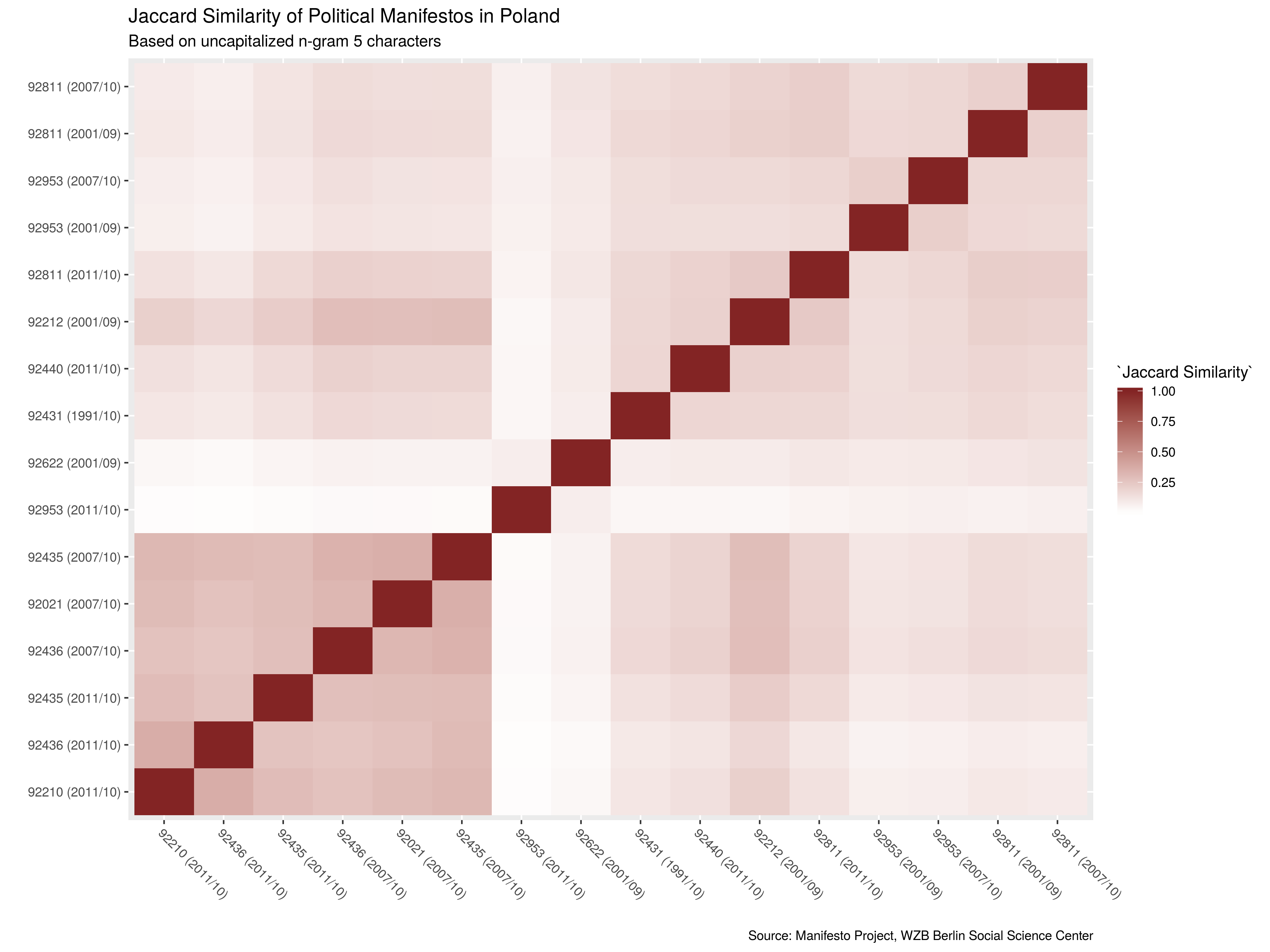

Supplement: Multimedia component 4 [file mmc4.zip › poland.png]

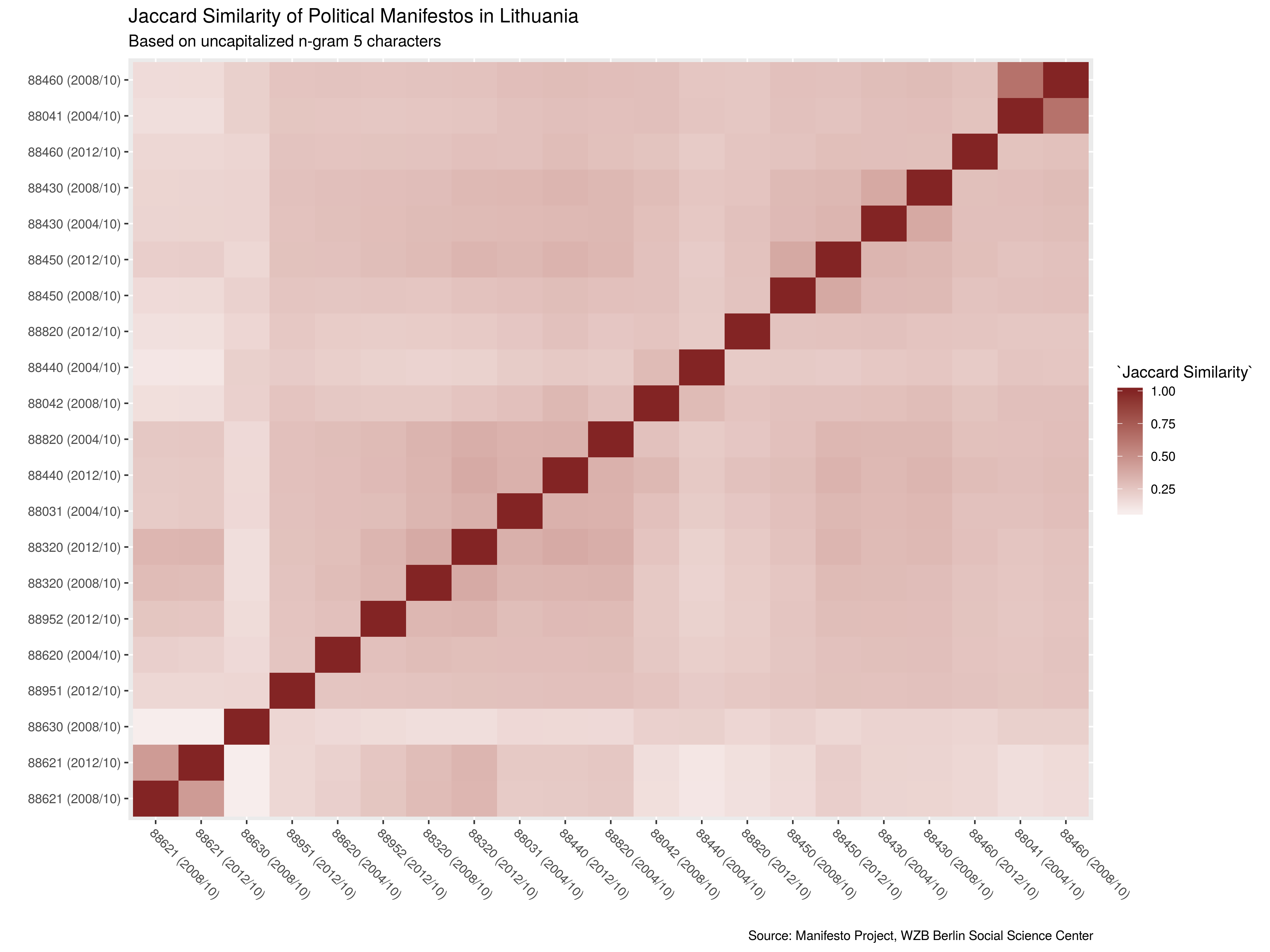

Supplement: Multimedia component 4 [file mmc4.zip › lithuania.png]

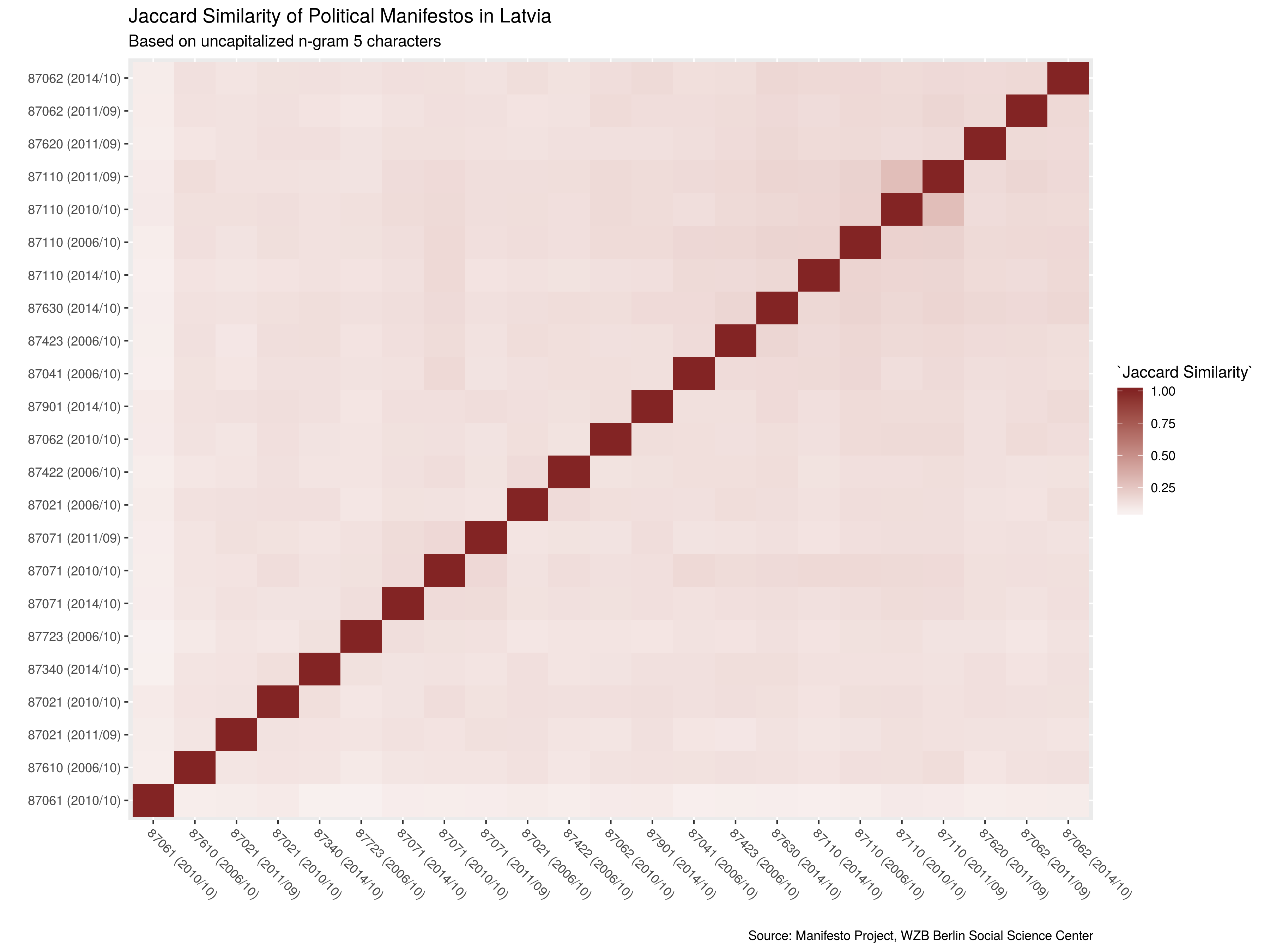

Supplement: Multimedia component 4 [file mmc4.zip › latvia.png]

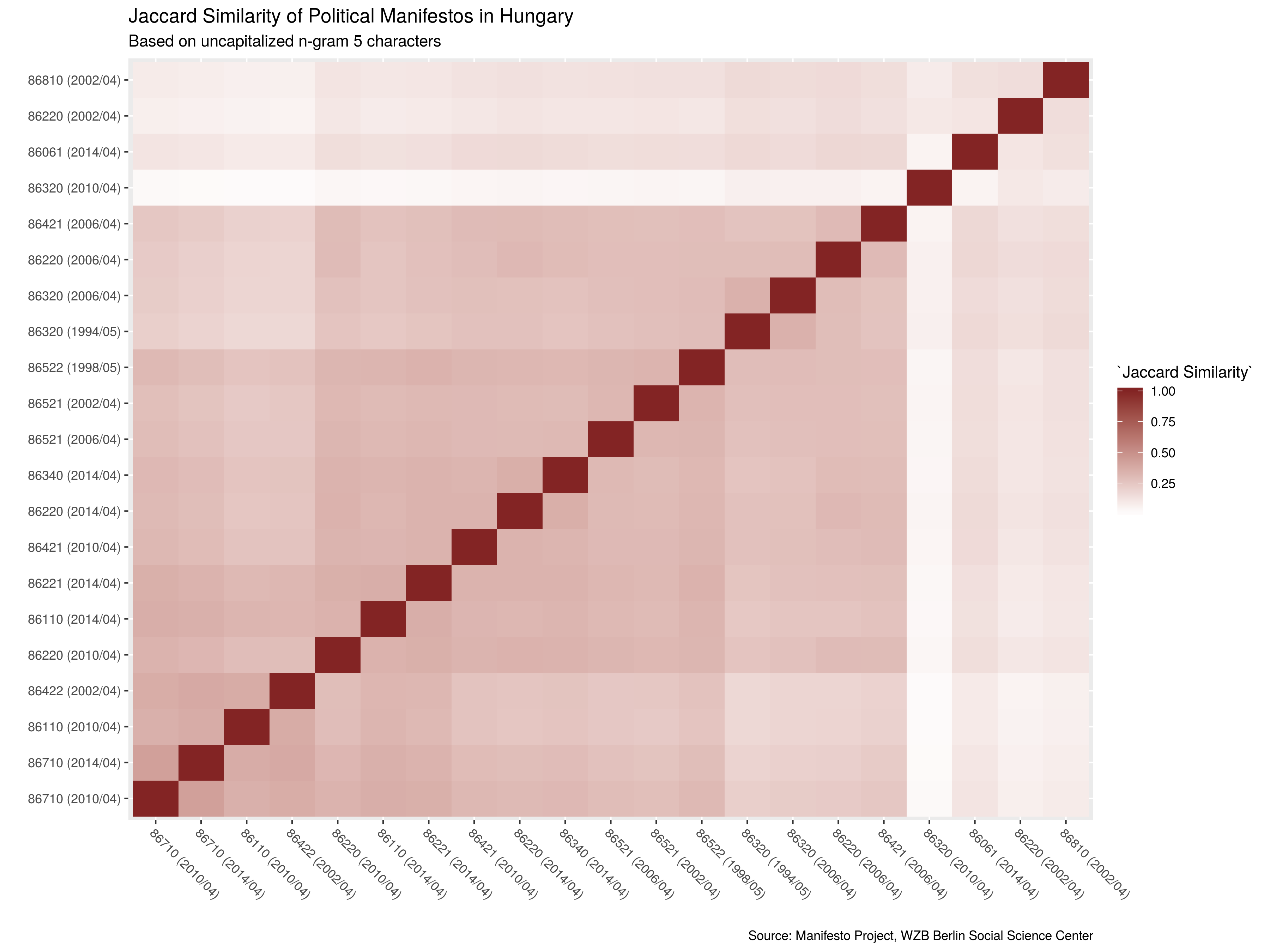

Supplement: Multimedia component 4 [file mmc4.zip › hungary.png]

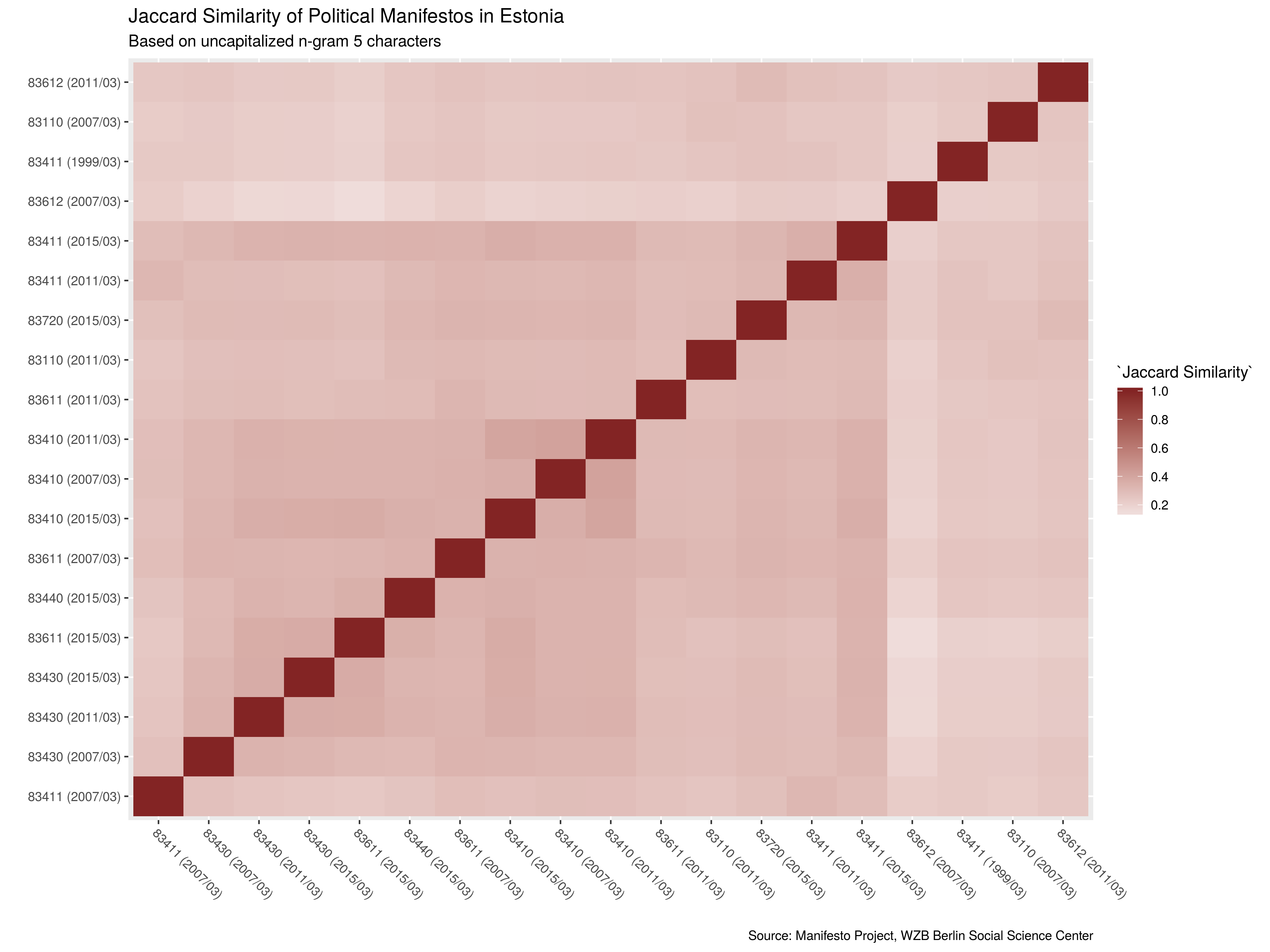

Supplement: Multimedia component 4 [file mmc4.zip › estonia.png]

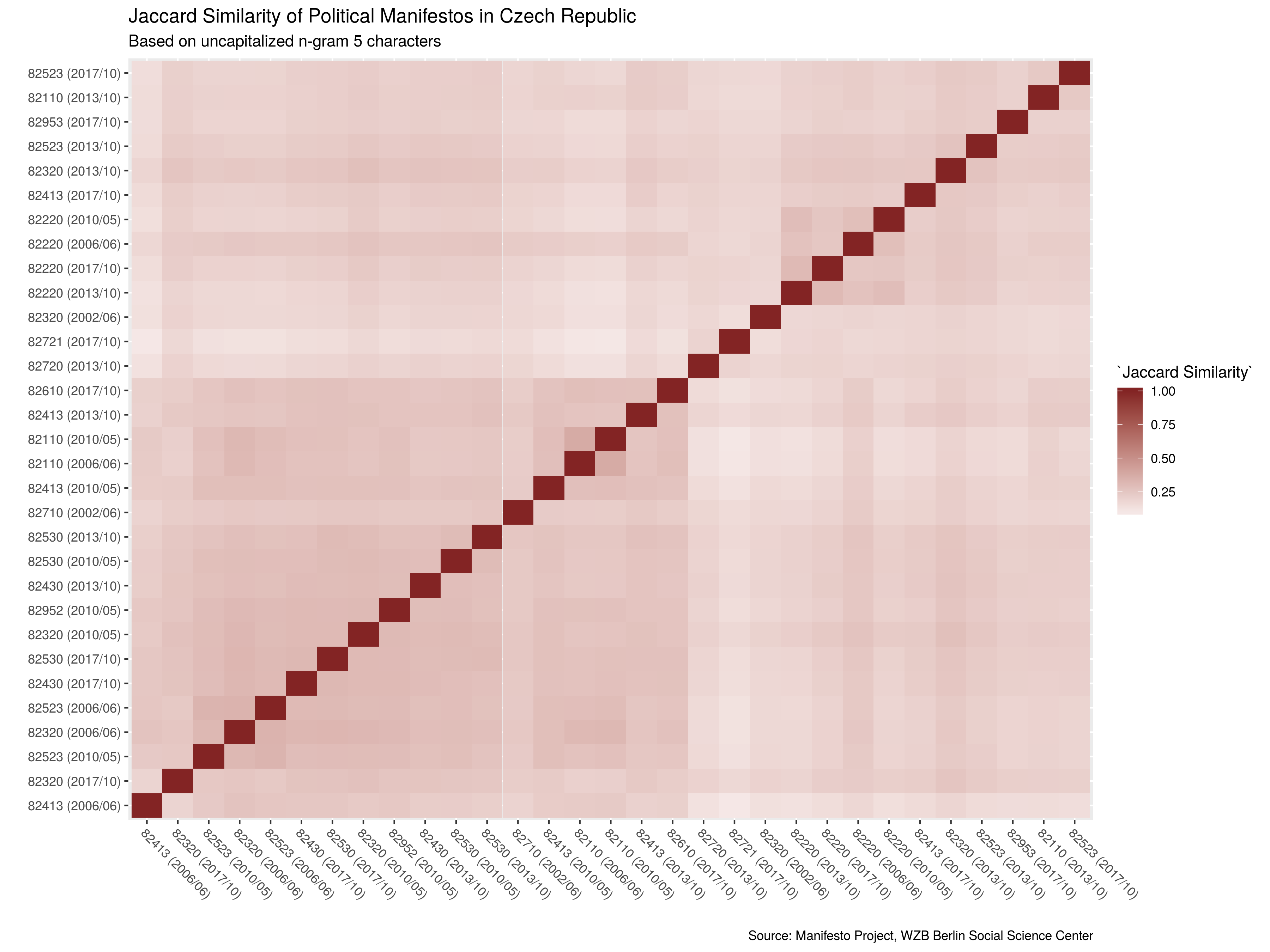

Supplement: Multimedia component 4 [file mmc4.zip › czechrepublic.png]

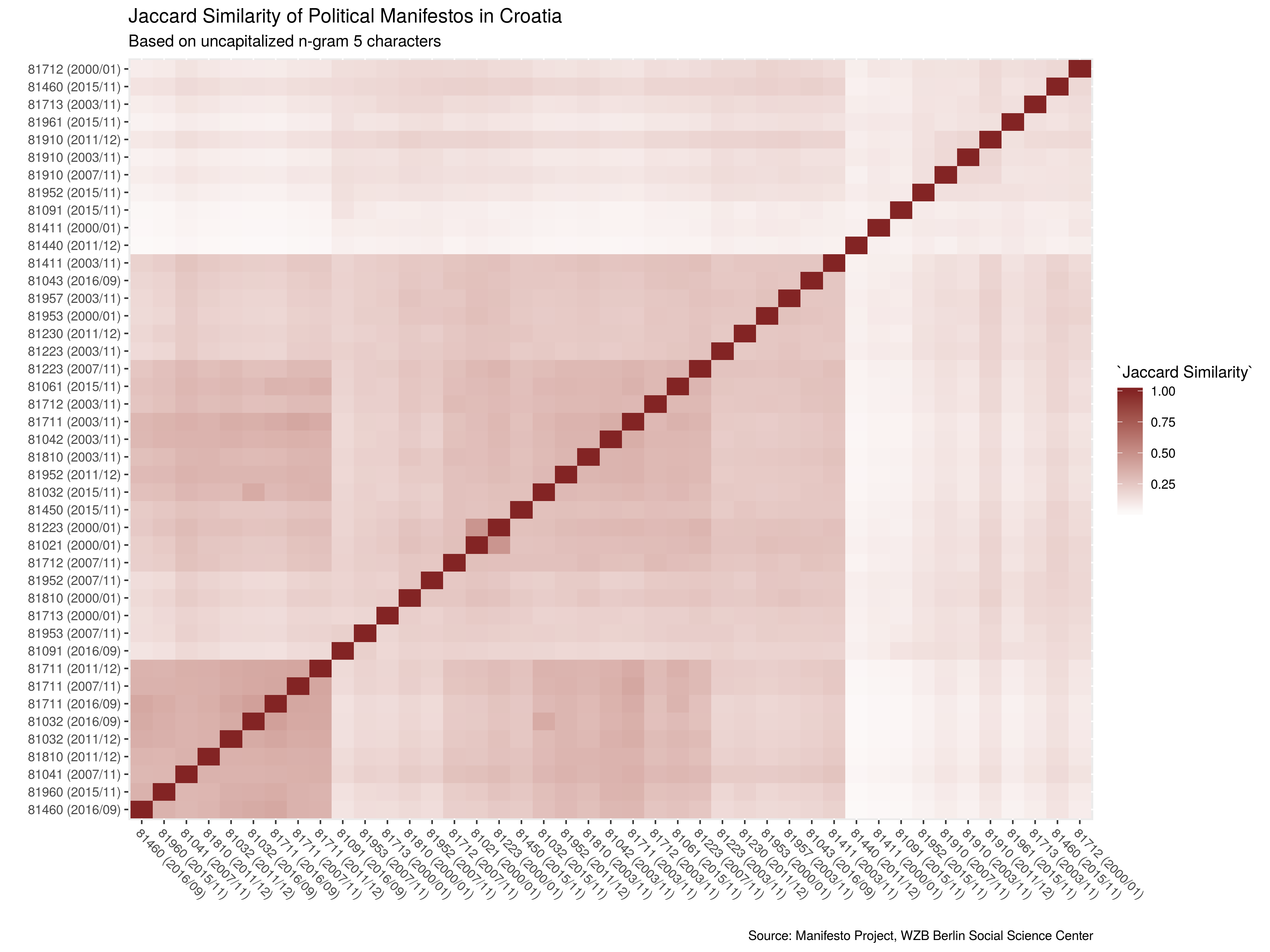

Supplement: Multimedia component 4 [file mmc4.zip › croatia.png]

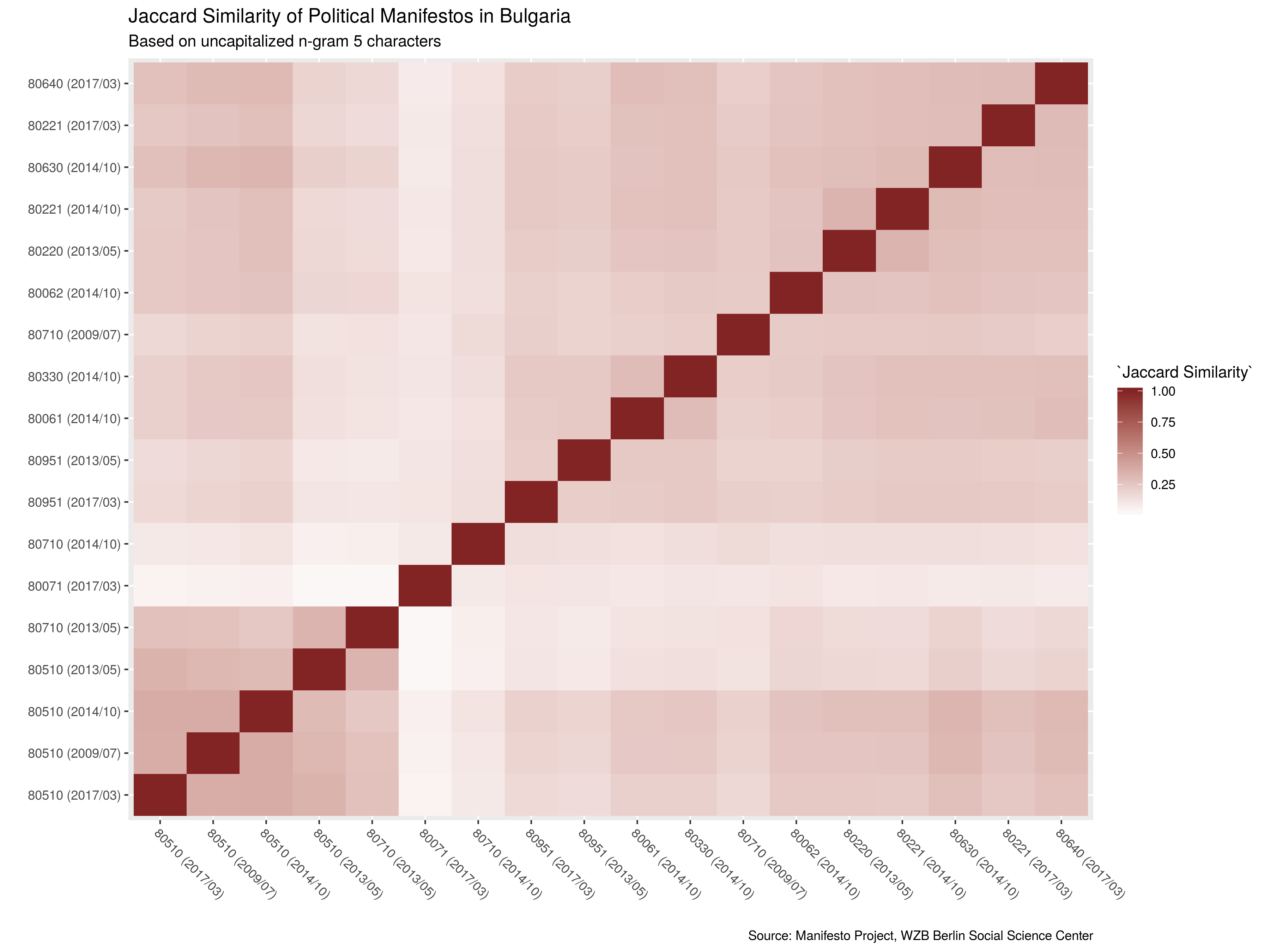

Supplement: Multimedia component 4 [file mmc4.zip › bulgaria.png]

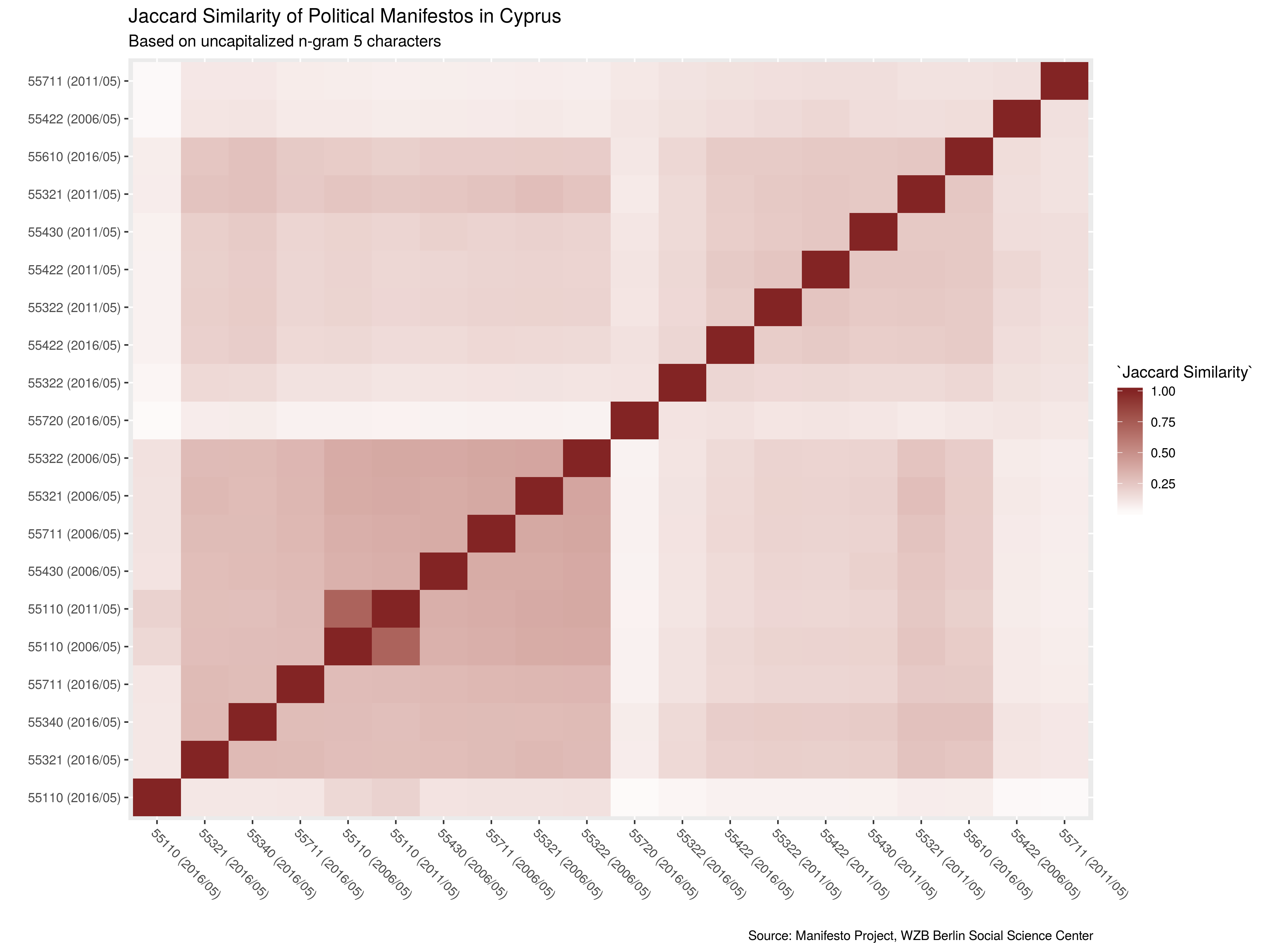

Supplement: Multimedia component 4 [file mmc4.zip › cyprus.png]

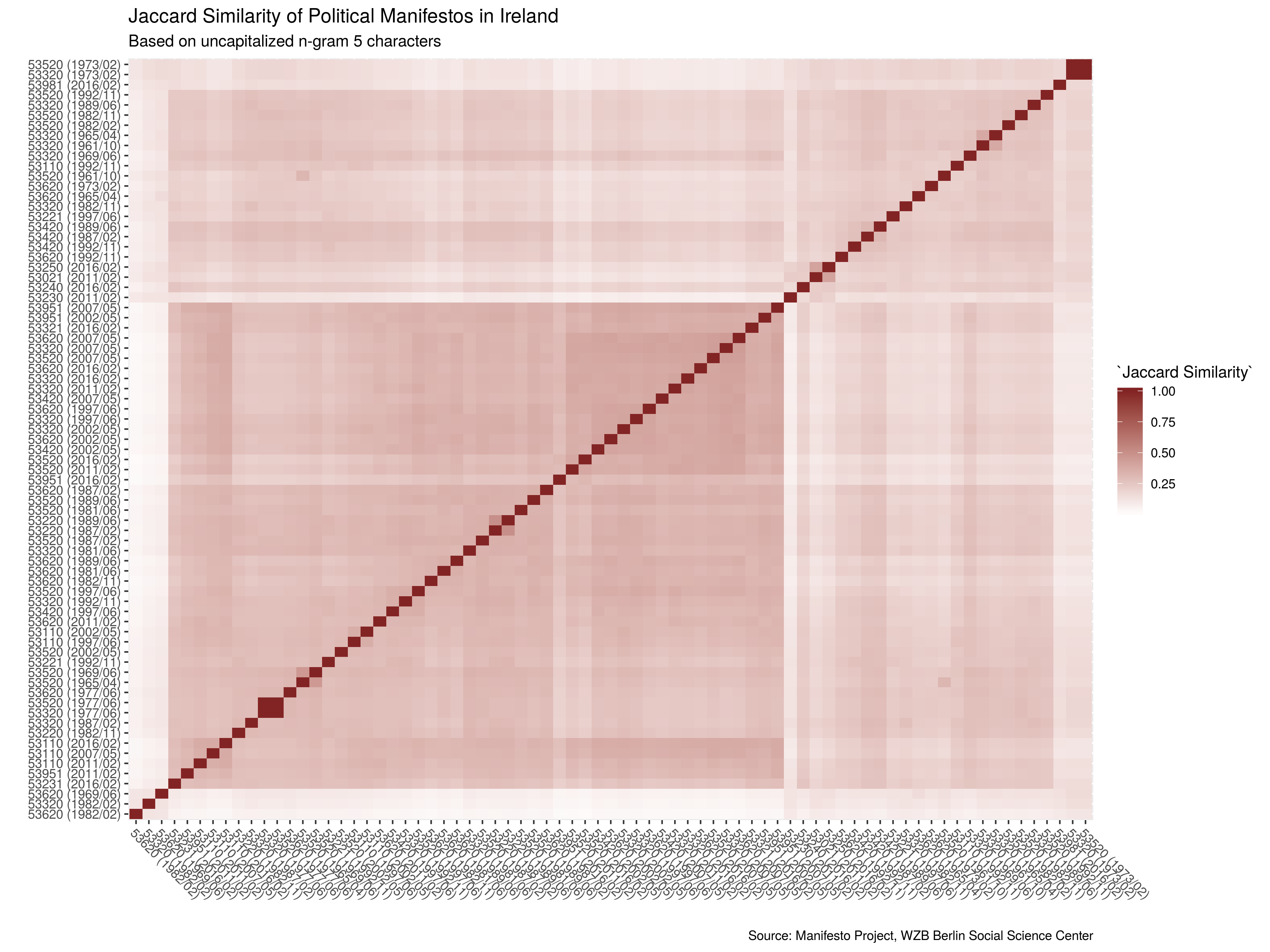

Supplement: Multimedia component 4 [file mmc4.zip › ireland.png]

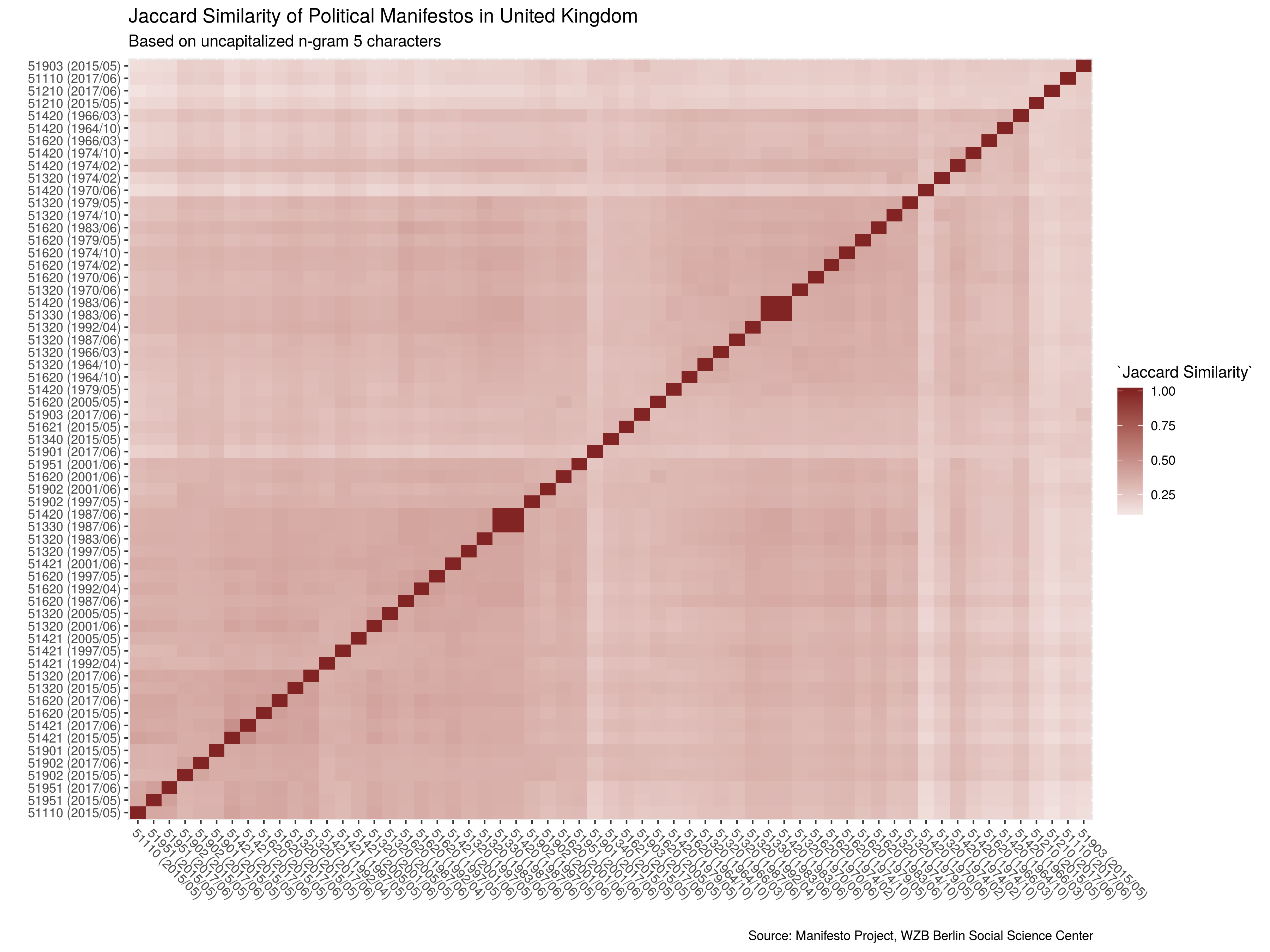

Supplement: Multimedia component 4 [file mmc4.zip › unitedkingdom.png]

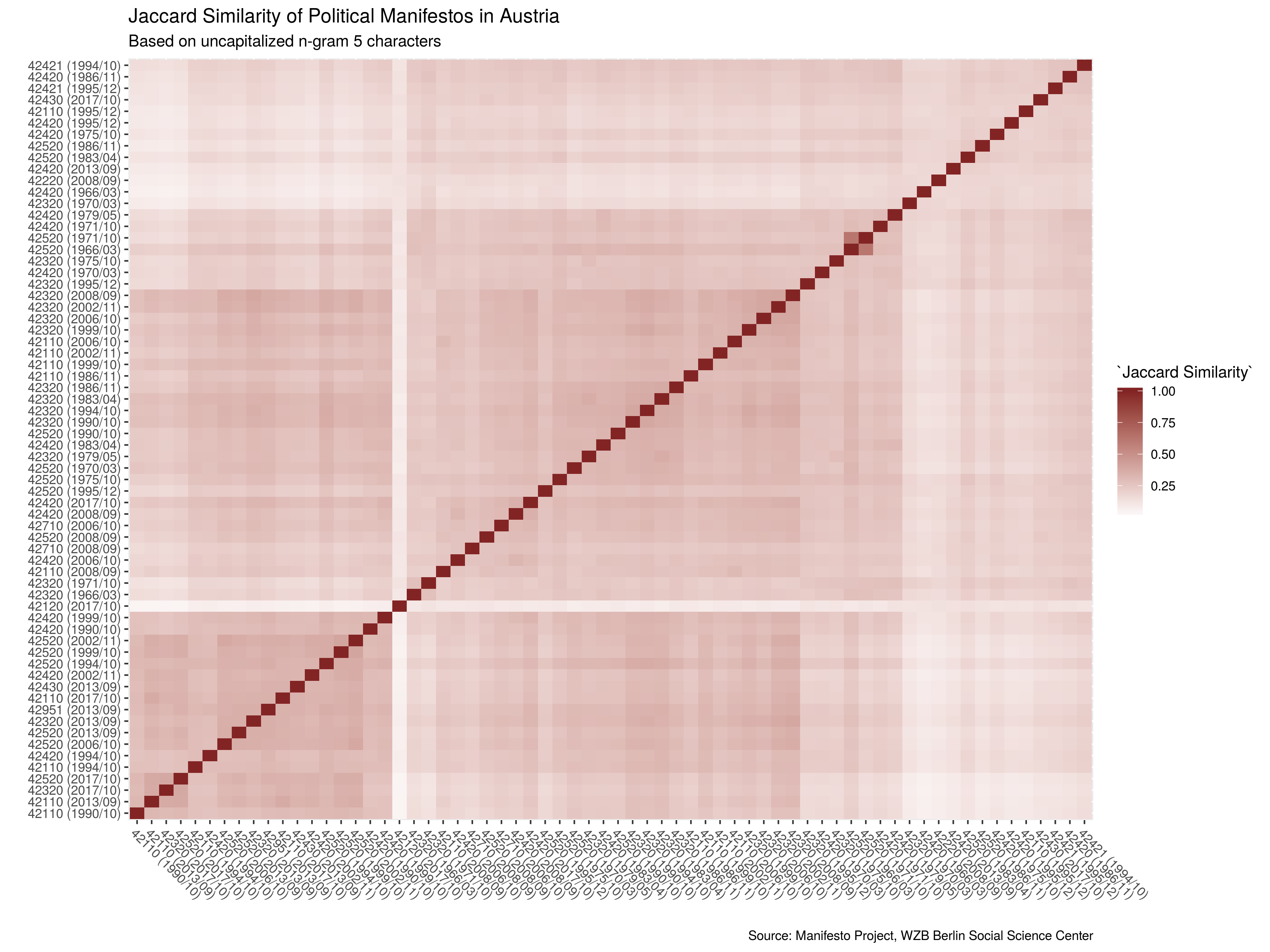

Supplement: Multimedia component 4 [file mmc4.zip › austria.png]

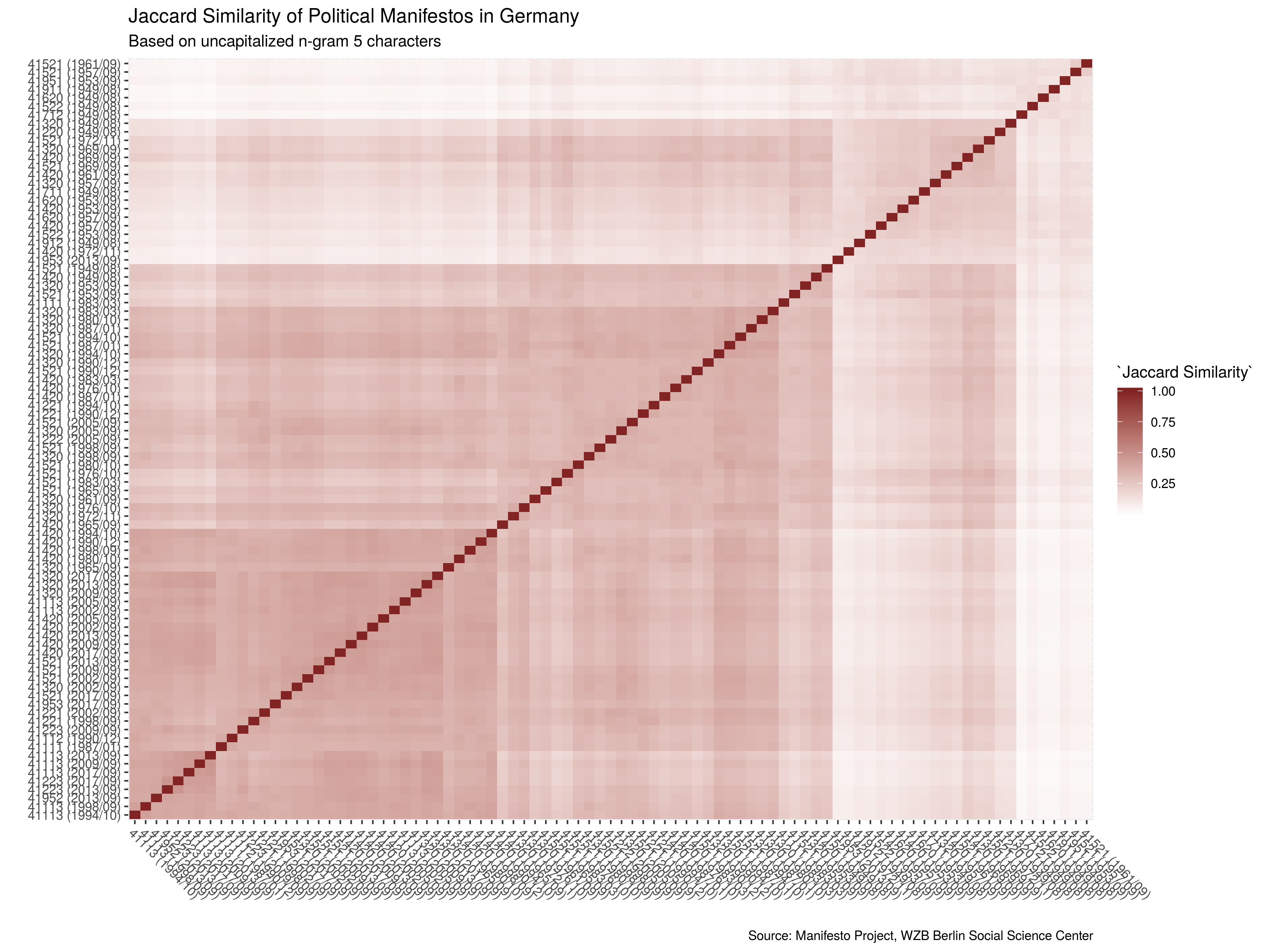

Supplement: Multimedia component 4 [file mmc4.zip › germany.png]

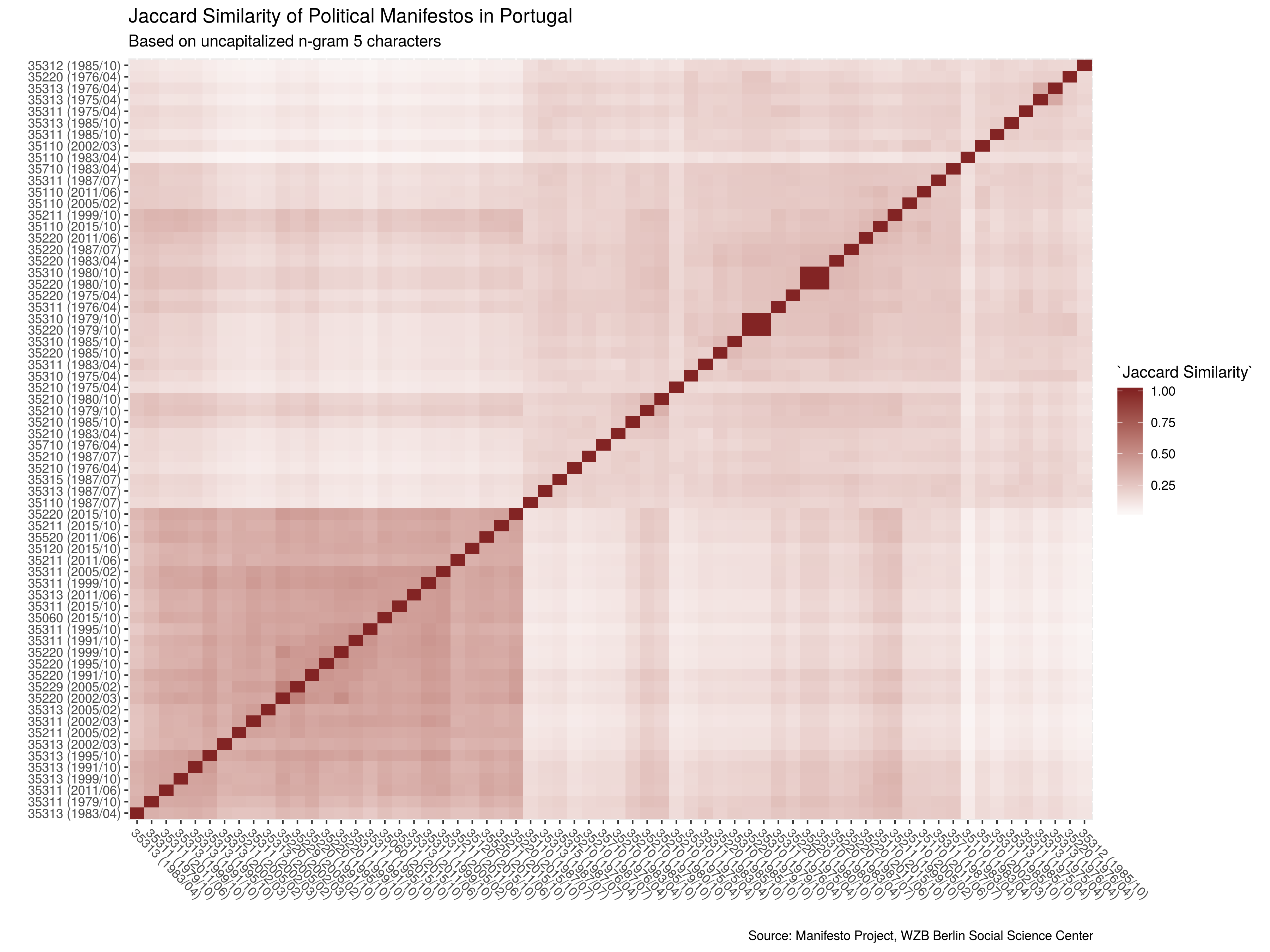

Supplement: Multimedia component 4 [file mmc4.zip › portugal.png]

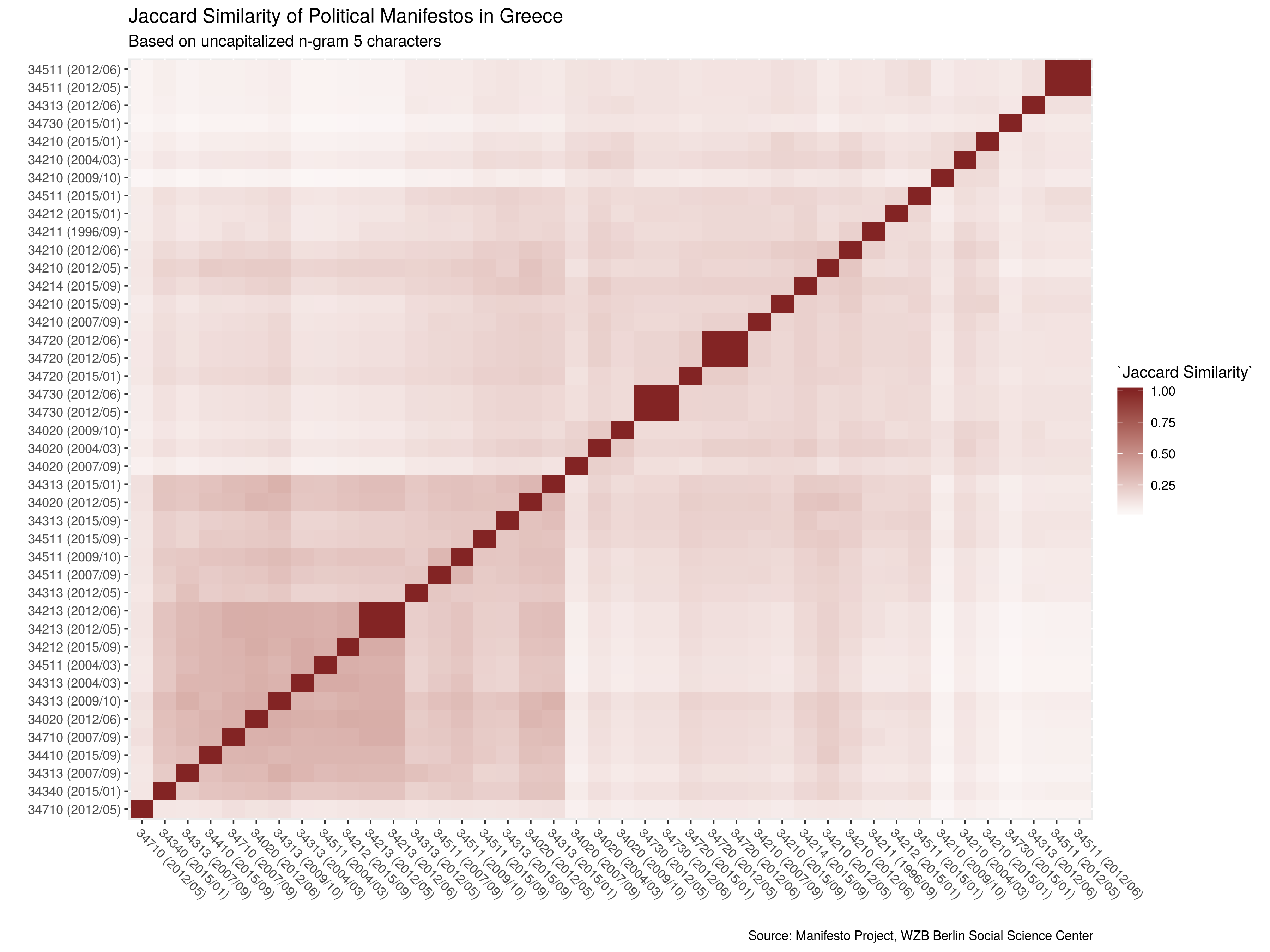

Supplement: Multimedia component 4 [file mmc4.zip › greece.png]

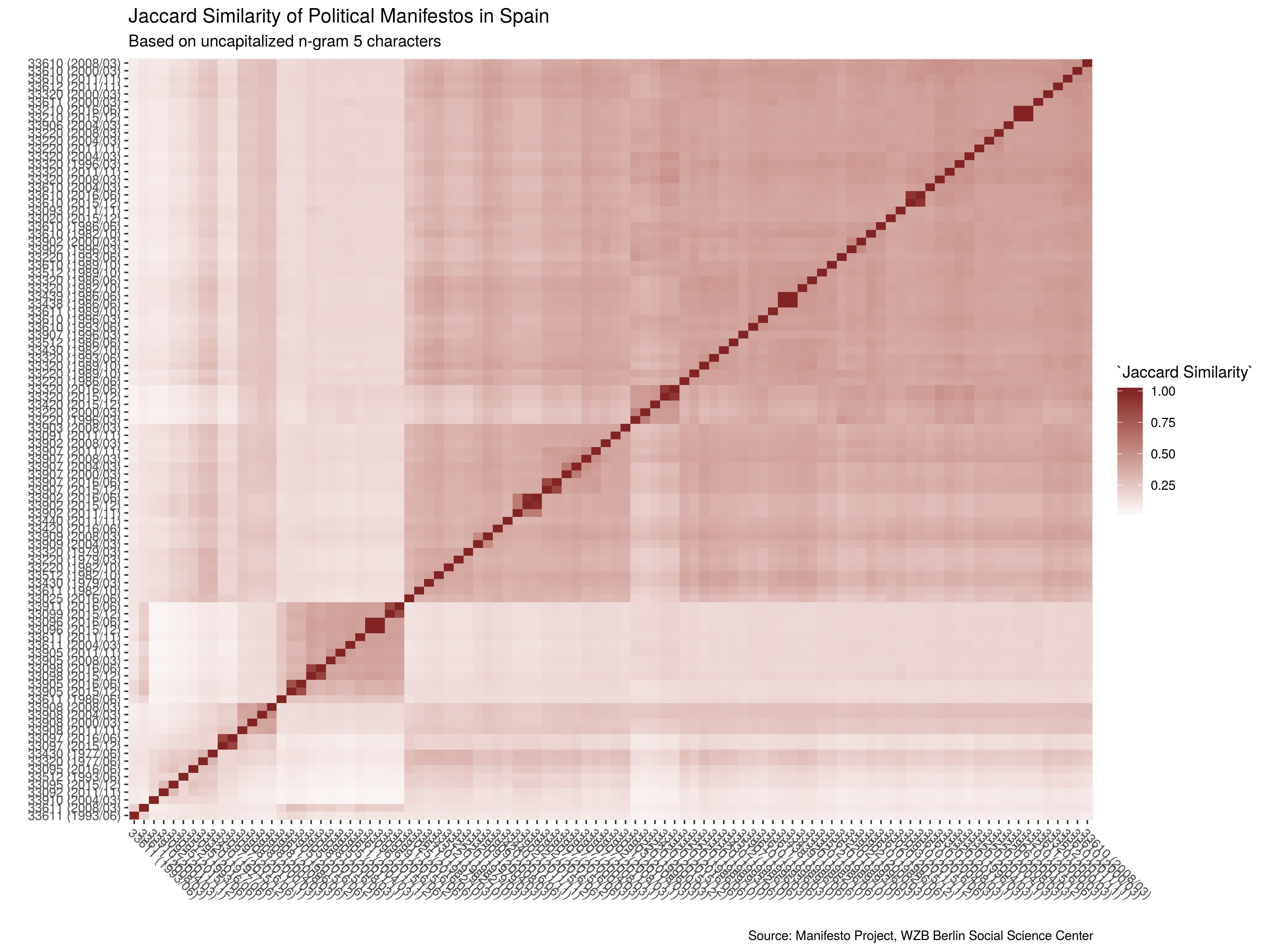

Supplement: Multimedia component 4 [file mmc4.zip › spain.png]

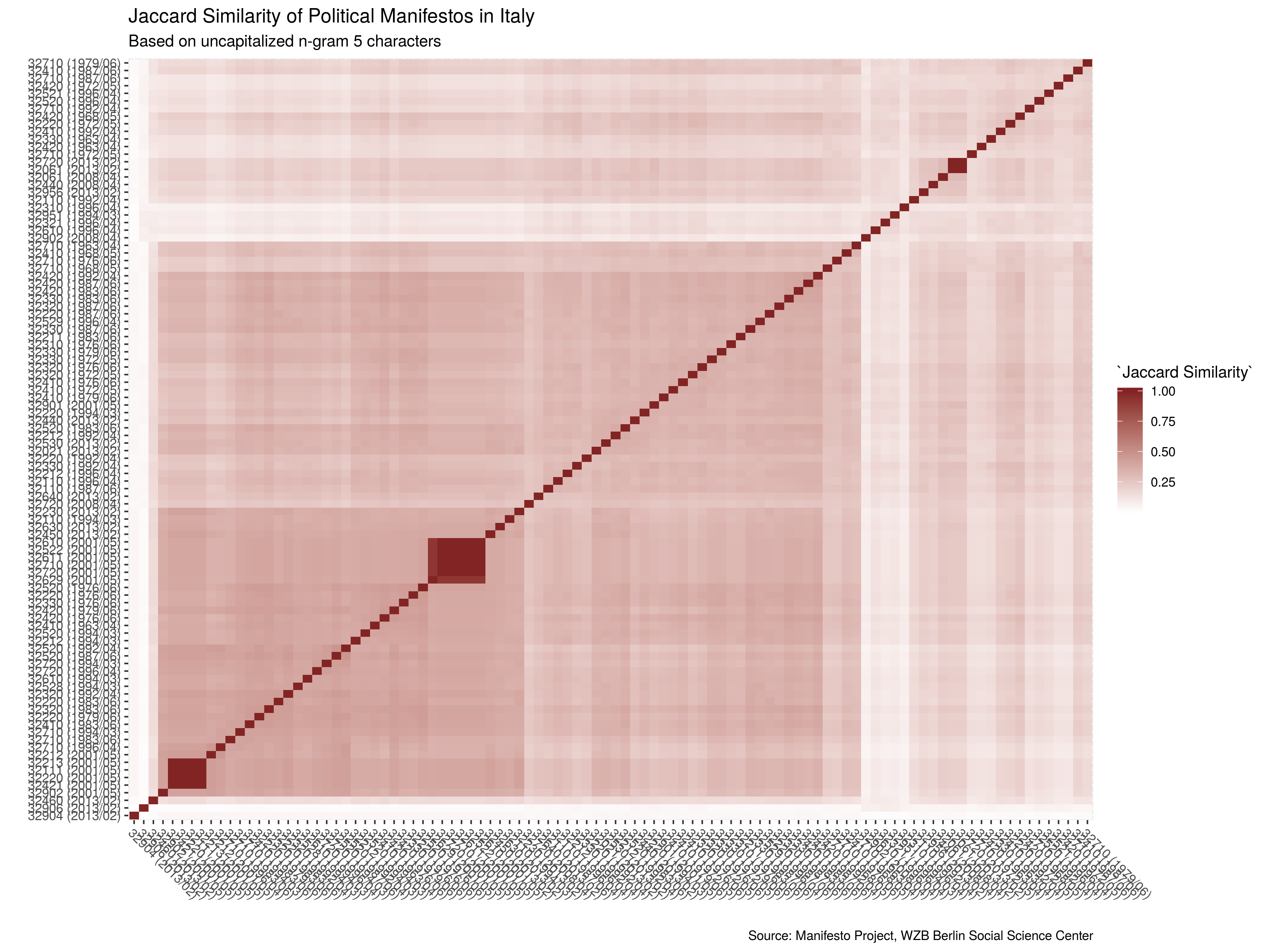

Supplement: Multimedia component 4 [file mmc4.zip › italy.png]

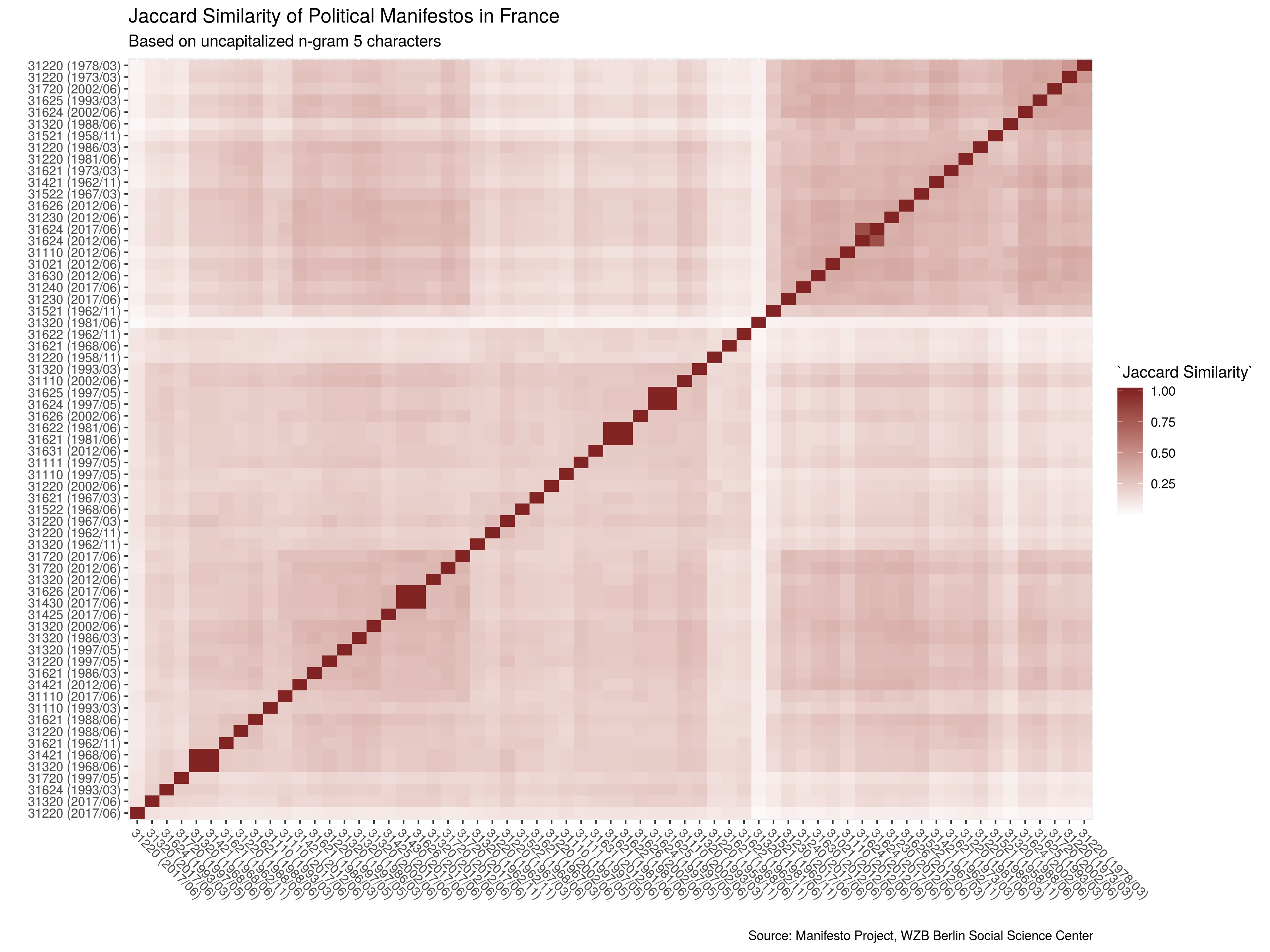

Supplement: Multimedia component 4 [file mmc4.zip › france.png]

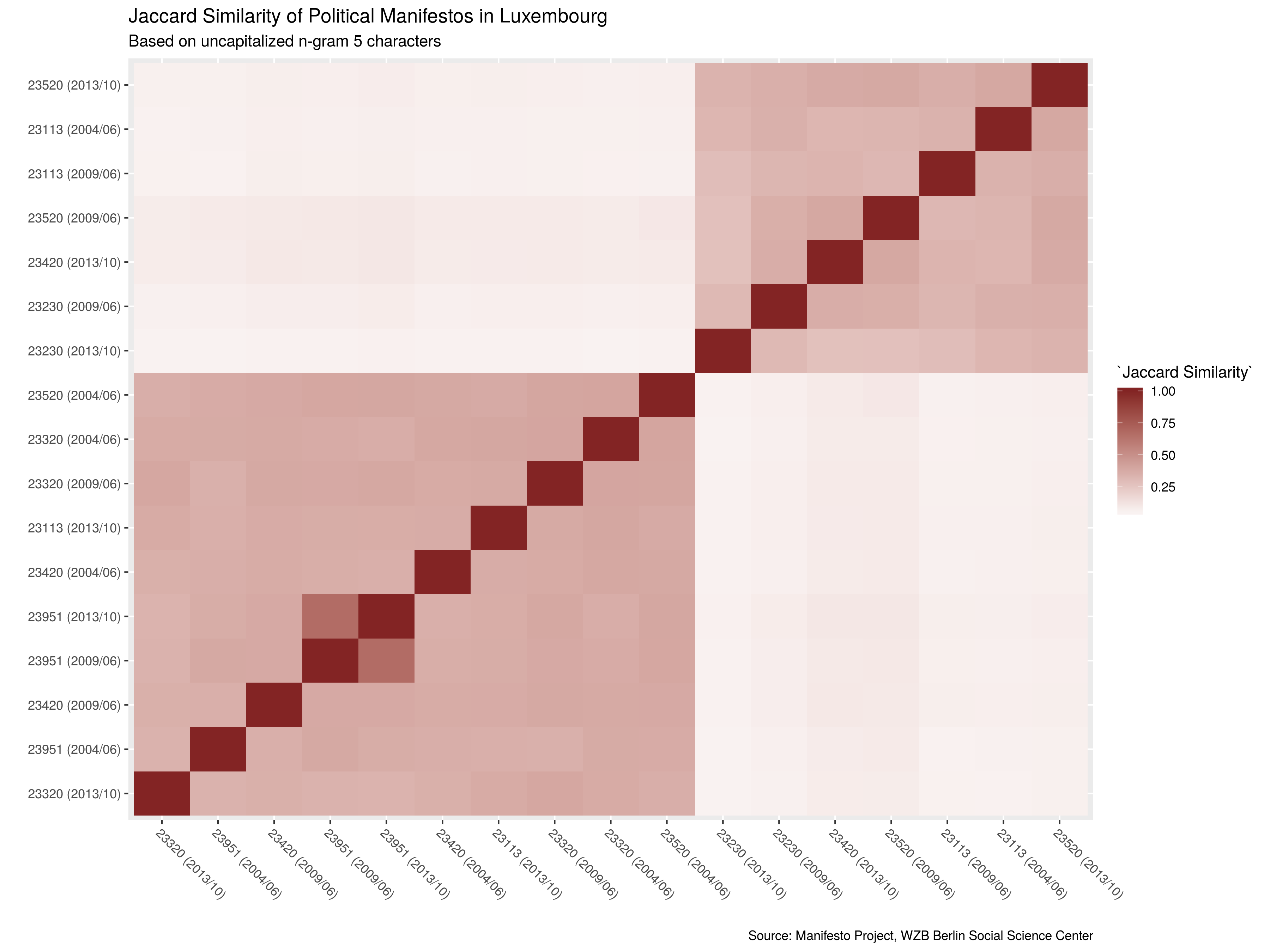

Supplement: Multimedia component 4 [file mmc4.zip › luxembourg.png]

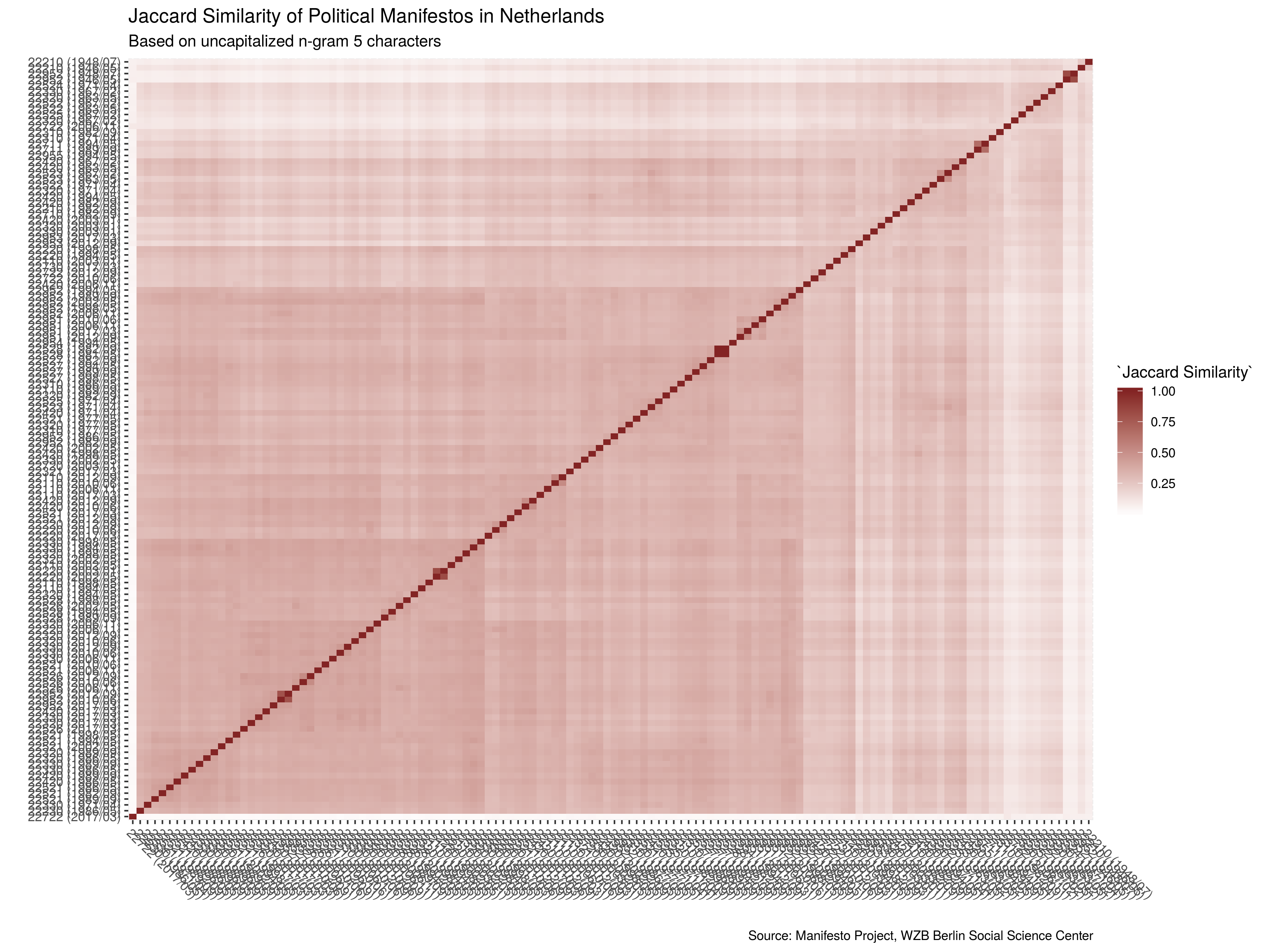

Supplement: Multimedia component 4 [file mmc4.zip › netherlands.png]

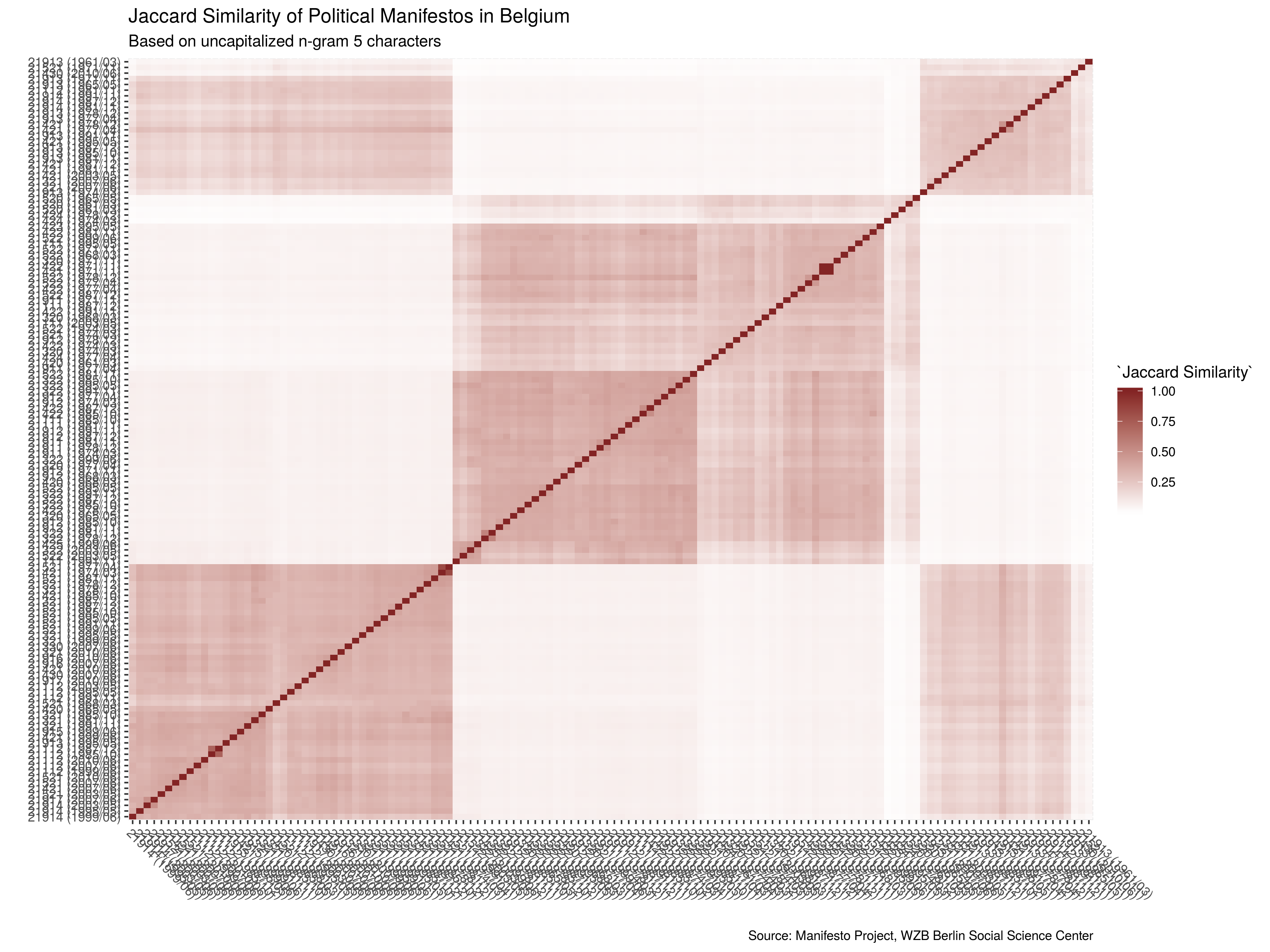

Supplement: Multimedia component 4 [file mmc4.zip › belgium.png]

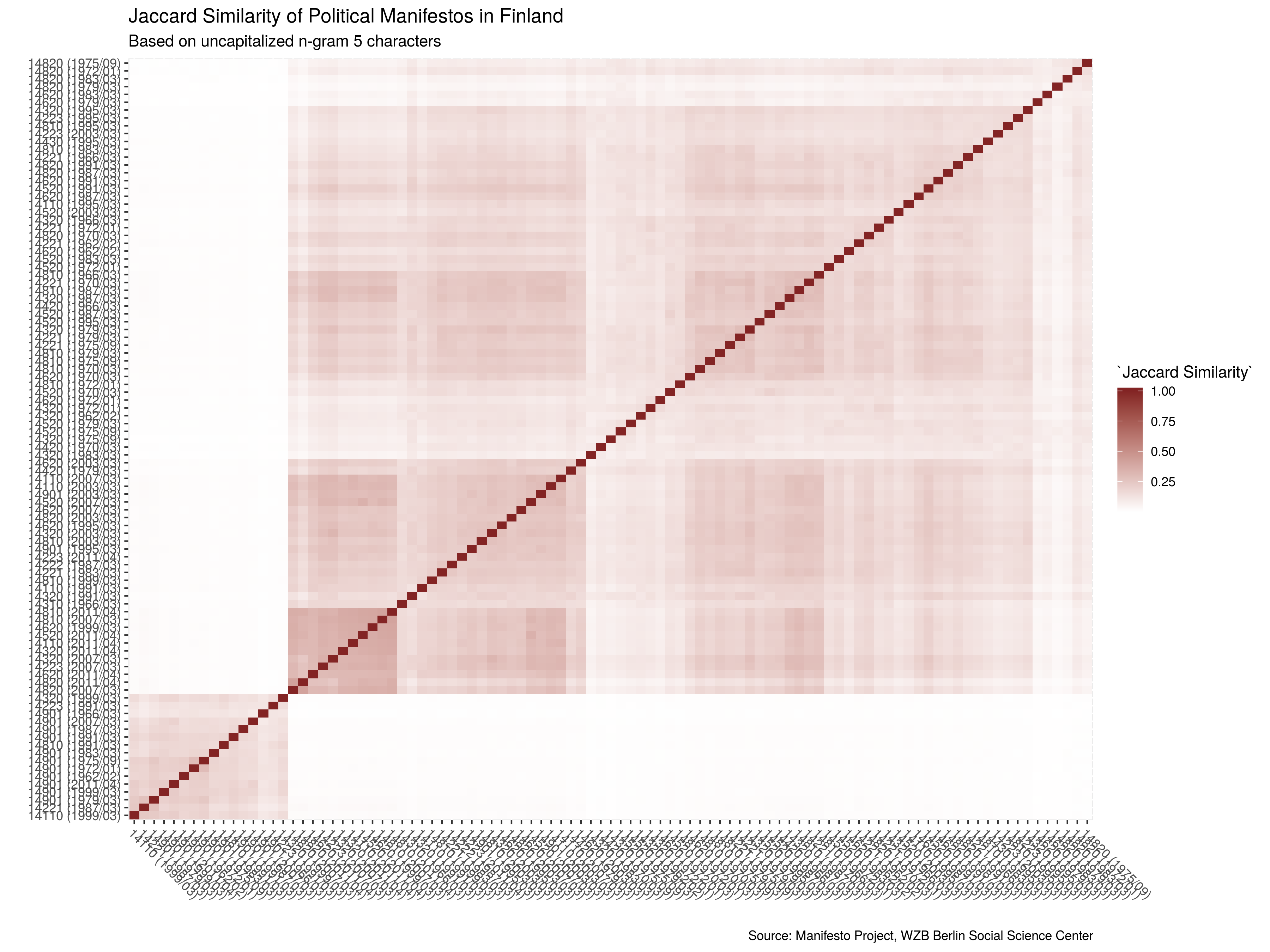

Supplement: Multimedia component 4 [file mmc4.zip › finland.png]

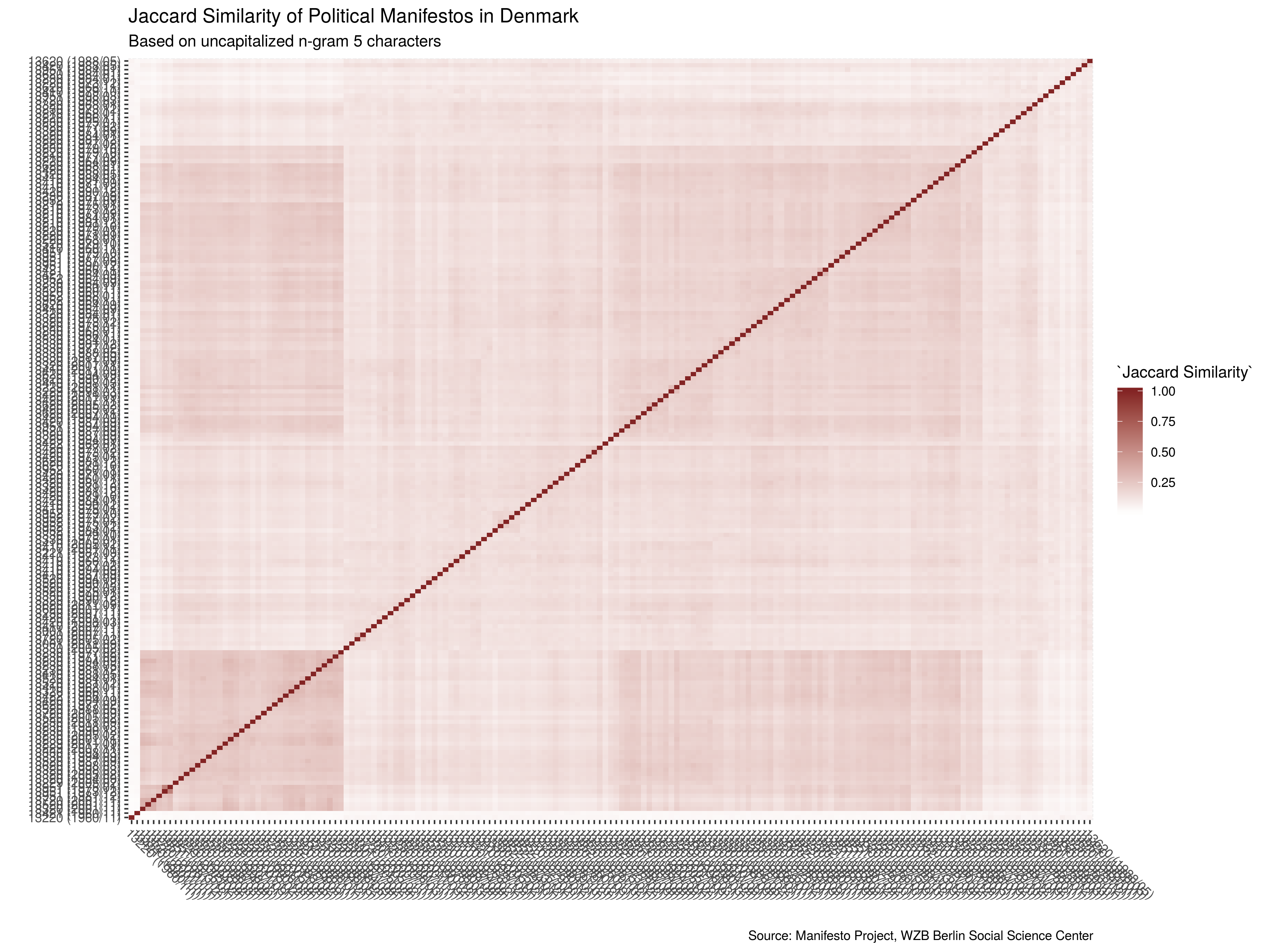

Supplement: Multimedia component 4 [file mmc4.zip › denmark.png]

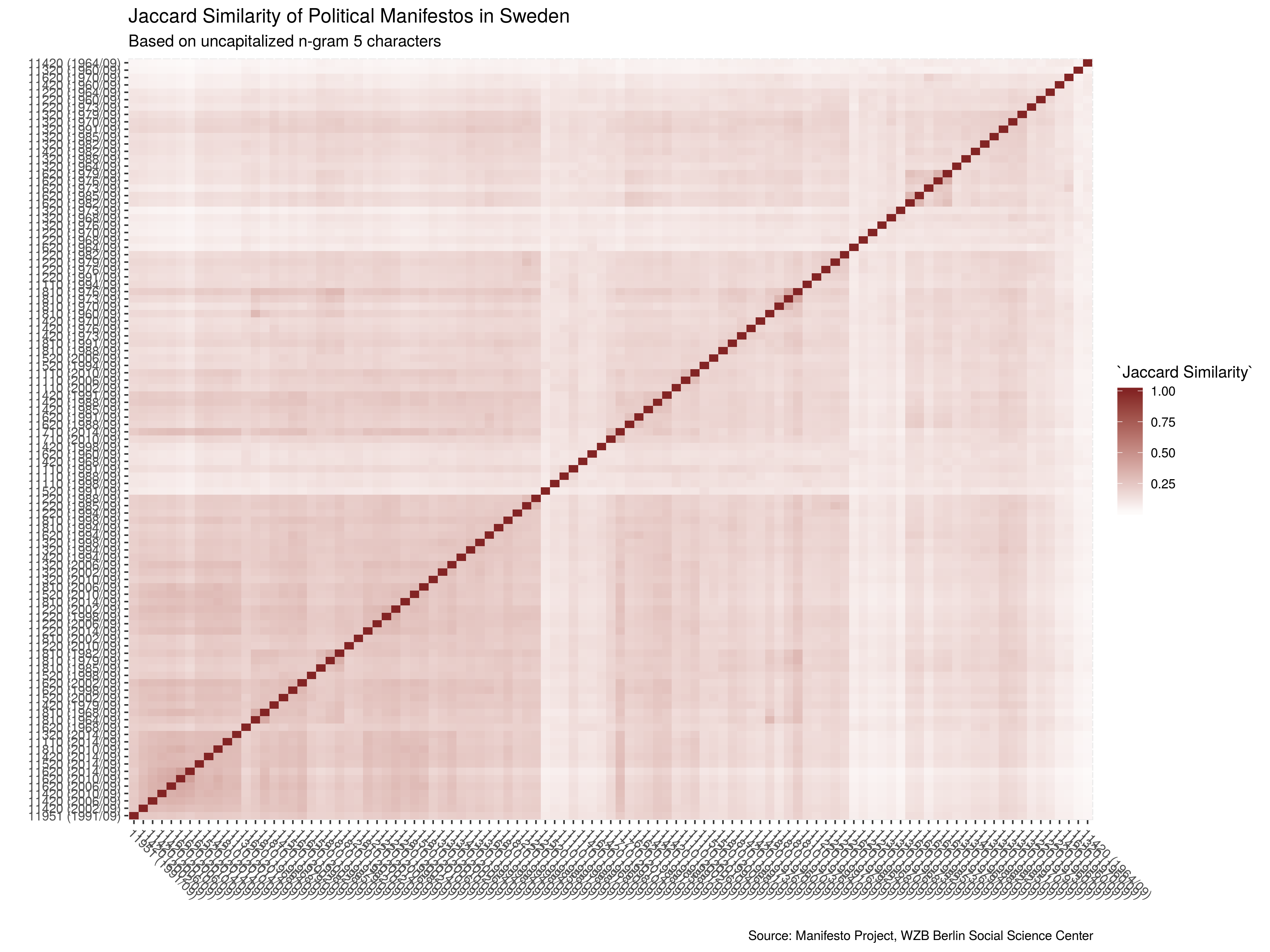

Supplement: Multimedia component 4 [file mmc4.zip › sweden.png]
